# The landscape of pregnancy and prepregnancy cohorts: a scoping review

**DOI:** 10.1186/s12884-025-07949-7

**Published:** 2025-10-28

**Authors:** Lauren Maxwell, Regina Gilyan, Sayali Arvind Chavan, Shaila Akter, Mahir Bhatt, Solveig A. Cunningham, Thomas Jaenisch

**Affiliations:** 1 Heidelberg Institute of Global Health, Universitätsklinikum Heidelberg, Im Neuenheimer Feld 130/3, Heidelberg, Germany; 2 Institute of Tropical Medicine and Public Health, Charité – Universitätsmedizin, Berlin, Germany; 3 Netherlands Interdisciplinary Demographic Institute, Lange Houtstraat 19, 2511 CV Den Haag, Netherlands; 4 Hubert Department of Global Health, Emory University, Atlanta, Georgia; 5 Center for Global Health, Colorado School of Public Health, University of Colorado, Denver, CO USA

**Keywords:** Scoping review, Pregnancy cohort, Prepregnancy cohort, Life-course epidemiology

## Abstract

**Background:**

Recent research in life course epidemiology has demonstrated the importance of evaluating how prepregnancy and pregnancy exposures affect later life developmental outcomes. In this scoping review, we identified and described completed or ongoing pregnancy and prepregnancy cohorts to assess gaps in the maternal exposures and child outcomes measured in these initiatives and inform future research investments.

**Methods:**

We developed a systematic search that included text and MeSH terms and was tailored for four biomedical citation databases. We applied the Arskey and O’Malley scoping review methodology. We selected a scoping review methodology to provide a comprehensive overview of pregnancy and prepregnancy cohorts and their characteristics. Two reviewers independently conducted the title, abstract, full-text screening, and data charting; a third reviewer resolved discrepancies. The results were summarised in narrative form.

**Results:**

We reviewed 147 manuscripts that presented findings from 56 pregnancy and two prepregnancy cohorts, 23 of which were ongoing. Half of the pregnancy cohorts were based in Europe. The most commonly described maternal exposures were nutrition, anthropometric measures, non-communicable diseases (NCDs), and demographic factors. Children’s mental, behavioural, neurodevelopmental, and physical outcomes were the most commonly measured outcomes. Fewer studies evaluated infectious disease, biomarkers, and environmental or workplace exposures. No cohorts examined vaccine or climate-related exposures during pregnancy. About half of the cohorts collected samples from pregnant women or the fetus, and a third from children, with blood being the most common sample type. Most studies did not indicate how data or samples could be accessed.

**Conclusions:**

This comprehensive overview of pregnancy and prepregnancy cohorts provides a foundation for cross-cohort coordination. Infectious disease, vaccine, environmental, and climate-related exposures and microbiome, immune function, and economic outcomes remain underrepresented in pregnancy and prepregnancy cohorts.

**Ethics and dissemination:**

This scoping review summarises findings from existing publications in peer-reviewed journals and did not require ethics review.

**Supplementary Information:**

The online version contains supplementary material available at 10.1186/s12884-025-07949-7.

## Background

Pregnancy and prepregnancy cohorts are essential for understanding the short- and long-term effects of pre- and pregnancy exposures to infectious disease (ID), nutrition, medication, vaccines, illicit drugs, and environmental, behavioural, and socioeconomic exposures. Pregnancy cohorts are longitudinal observational studies that enroll women during pregnancy and follow them through gestation, childbirth, and, in some cases, into the postpartum period and early childhood, tracking maternal exposures during pregnancy and their potential effects on pregnancy outcomes and fetal, infant, and child health. Prepregnancy cohorts recruit participants and begin data collection on various exposures before conception, but otherwise follow a similar design, tracking maternal, fetal, and child outcomes. Pregnant women are underrepresented in or excluded from most clinical trials, which underscores the need for observational data collected during pregnancy to understand the effects of medication or vaccine exposures. Prepregnancy cohorts offer unique insights into how the preconception period affects fetal, infant, and child development, providing information on the potential risks and benefits associated with various exposures.

### The importance of prepregnancy and pregnancy cohorts with longer-term follow-up

Many adverse effects of prenatal exposures, including cognitive, behavioral, and neurodevelopmental outcomes, cannot be reliably assessed before two years of age. Key developmental milestones in executive function, language, and social-emotional regulation typically begin to manifest between 18 and 36 months of age and require age-appropriate standardized assessments that are not feasible in neonates or infants [1, 2]. Neurodevelopmental delays associated with exposures such as maternal obesity [3], malnutrition [4, 5], diabetes [6], hypertension [7], and infection [8] often do not present until after the second year of life, when children are more capable of completing structured cognitive and behavioral evaluations. For example, impairments linked to prenatal exposure to Zika virus or cytomegalovirus may include delayed language acquisition, hearing loss, or motor impairment [8], which cannot be detected reliably at birth or during early infancy. Additionally, pregnancy-related exposures are linked to development of mental health issues [9], cancers [10], and other non-communicable diseases (NCDs) that may not develop before two years of age. Cataloguing pregnancy and prepregnancy cohorts that track infants to two years and beyond helps to highlight the available data that can be used to examine such associations further. Below, we briefly describe key maternal exposures and their potential short- and long-term effects on child health, underscoring the importance of prepregnancy and pregnancy cohort studies in advancing our understanding of fetal, infant, and child health.

### Nutrition

Maternal nutritional intake can have short and long-term effects on child health and disease risk [5, 11]. Maternal obesity has been linked to perinatal mortality [12], impaired child glucose tolerance [13], and poor cognitive performance [3]. Maternal underweight is related to a higher risk of low birth weight (LBW), under-five mortality, and poor physical and psychological development [14]. Nutrient deficiencies in pregnancy can stunt fetal growth, increasing the likelihood of LBW and small-for-gestational-age (SGA) [11]**.** Maternal underweight and malnutrition increase the child’s risk of congenital heart disease (CHD) [15] and NCDs [11]. Studies have also related maternal choline and vitamin D deficiency to impaired cognitive development [4, 5]. Given such wide-ranging consequences of maternal nutrition, pregnancy and prepregnancy cohorts provide a critical window to identify nutritional exposures before and during pregnancy, and enable the examination of their effects on maternal and child health (MCH).

### Non-communicable diseases

Maternal NCDs, such as obesity, diabetes, hypertension, cardiovascular disease, and autoimmune disorders, can influence fetal development through a number of pathways, increasing the risk of adverse birth outcomes and long-term health complications in the offspring. For example, maternal hypertension was linked to adverse child growth outcomes and a heightened risk of cardiovascular disease (CVD) [16, 17]. Maternal diabetes is associated with higher child body-mass index (BMI) [18, 19], psychiatric disorders [9], and delayed language development [6].

### Infectious exposures

Pregnant women with COVID-19 [20, 21], symptomatic dengue [22], malaria [23], Zika virus (ZIKV) [24], syphilis [25], parvovirus B19 [26], rubella [27], cytomegalovirus, and herpes simplex virus (HSV) [28] have a higher risk of neonatal morbidity and mortality. Maternal infections during pregnancy have been related to a higher risk of later life developmental [29], psychological [30–32], and physical issues, including cancers [10]. Both ZIKV and rubella infections in pregnancy are related to a spectrum of adverse congenital outcomes, such as cardiac, ocular, and hearing defects [27], as well as neurological abnormalities [33, 34]. ZIKV is also a recognised cause of long-term impairments, such as cerebral palsy and epilepsy [35], while rubella has been associated with later-life conditions, including diabetes, thyroid disease, growth hormone deficiency, and progressive panencephalitis [36]. Malaria [23], HSV [37], CMV [38], and ZIKV [39] infection in pregnancy have been linked to neurodevelopmental issues that are not observable at birth, including neurocognitive and language delays, and later-life hearing loss.

### Social exposures

Social determinants, such as socioeconomic position, education, employment, and living environment play a critical role in shaping maternal, fetal and child health. Low socioeconomic status is consistently associated with adverse pregnancy outcomes, particularly low birthweight and preterm birth [40]. In addition to socioeconomic factors, minority race identity has been linked to adverse pregnancy outcomes such as higher rates of morbidity and mortality [41], while exposure to poverty during pregnancy is associated with delayed cognitive development and poor educational outcomes in children [42]. Maternal exposure to intimate partner violence (IPV) has also been linked to poor developmental outcomes, partly through its effect on maternal depression [43, 44]. While this body of evidence continues to grow, interventions to mitigate the effects of social disadvantage remain limited, and few studies evaluate their effectiveness across diverse settings.

### Natural disasters or societal crises

Prenatal exposure to disasters may affect child development and physical and mental health through its impact on maternal stress, although a recent systematic review showed conflicting findings, with some studies identifying strong associations – such as between famine and schizophrenia – while others found limited or no effects or showed inconsistent results across similar contexts [45]. Prenatal exposure to famine is associated with children’s risk of CVD, Type 2 diabetes mellitus (T2DM), cancer [46], mental health disorders [47], and reduced quality of life in adulthood [48].

### Study approach and objectives

Exposures immediately before or during fetal development are essential determinants of childhood growth and development and the risk for disease later in life [49]. In this scoping review, we systematically identified pregnancy and prepregnancy cohorts that followed children to at least two years of age to understand what populations, exposures, outcomes, and samples were included in these studies. We restricted our review to pregnancy and prepregnancy cohorts that followed children for at least two years to ensure inclusion of studies capable of capturing developmental outcomes that only emerge beyond infancy. Additionally, while there are many more cohorts that follow pregnant women to birth or until their infants are under age two, the magnitude of infrastructure, personnel, data, and biospecimen collection resources required to sustain longitudinal follow-up beyond two years is substantially greater than that needed for studies limited to birth outcomes or early infant development. Cohorts with extended follow-up provide rich insights into life course epidemiology and longitudinal causal inference [49]. By focusing on cohorts with follow-up of at least two years, our review prioritizes initiatives with the scope and scale necessary to examine how prepregnancy and maternal exposures influence long-term child development.

Information on data and biospecimen collection and availability can help researchers better utilise existing resources to understand the relation between maternal exposures and child physical and developmental outcomes. We also summarised information on pregnancy and prepregnancy cohort data sharing and registries and platforms to help researchers understand how cohort datasets and samples are made available for reuse.

Unlike systematic reviews, which focus on synthesizing findings from studies to answer a specific research question, scoping reviews aim to explore the existing literature and map the breadth of available research to help identify more specific subject areas for a subsequent systematic review. We chose a scoping review approach because we were interested in describing the ecosystem of pre- and pregnancy cohorts rather than summarising the findings of individual studies. Review questions were developed through consultation with the research team and feedback from biomedical researchers who work with pregnancy and prepregnancy cohorts in the context of ID and NCDs. Key review questions included:

1. What pregnancy and prepregnancy cohorts exist that follow infants to two years of age and beyond?
2. What exposures do they measure before or during pregnancy?
3. What maternal, fetal, infant, child, or adult outcomes do they measure?
4. What samples do they collect from pregnant women, their partners, the fetus, the infant, or the child?
5. Do they share participant-level data and samples from their cohort? Where? What mechanisms are in place to facilitate access to that data?

## Methods

We used the Arksey and O’Malley approach to scoping reviews [50] and presented results per the PRISMA Extension for Scoping Reviews guidance (PRISMA-ScR) [51]. The detailed PRISMA-ScR checklist is provided in Additional File 1. The protocol and systematic search strategy were published on F1000 before initiating the search [52].

### Data sources, search strategy, & eligibility criteria

We searched the following databases from 1 January 2000 to 19 February 2021: Ovid (Medline), Ovid (EMBASE), Web of Science, and LILACs. The search strategies (Additional File 2) included a combination of text and MeSH terms tailored for each database and were developed in consultation with an information scientist at the University of Colorado. We did not limit the search by geography or language. We included prospective, primary research studies that reported on pregnancy or prepregnancy cohorts and followed infants for more than two years after birth because of the importance of studying the impact of pre and pregnancy exposures on longer-term development. Prepregnancy cohorts were defined as cohorts that enrolled women and collected data before conception. We excluded retrospective cohorts limited to data from previously collected medical records or national registries that did not prospectively enroll participants before or during pregnancy. Cohorts that prospectively enrolled women prior to or during pregnancy that also included data from previously collected EHR were included. We excluded RCTs, commentaries, editorials, systematic or scoping reviews, non-peer-reviewed research, and non-pregnancy-focused longitudinal studies that did not assess pregnancy exposures and child outcomes.

### Title abstract, full-text screening, and data extraction

Citations were exported to EndNote X9 for deduplication. Two reviewers screened all titles, abstracts, and full texts. Disagreements during the title-abstract and full-text screening were resolved through discussion with a third reviewer. A list of studies excluded at the full-text review stage and reasons for exclusion is provided in Additional File 3. We developed and piloted a data extraction and charting form (Additional File 4) to facilitate the descriptive synthesis of our findings before beginning the charting process. Two reviewers independently completed data extraction and charting using COVIDENCE systematic review software [53]. At the beginning of the data extraction process, the two reviewers reviewed one another’s work to ensure consistent interpretation of data extraction and charting items. After a satisfactory level of agreement was achieved in the first three studies, they independently extracted data and reviewed any differences in the interpretation of the data after extraction was finalized for the remaining studies. Where studies presented data from the same cohorts, we summarised the results at the cohort level. To ensure the comprehensiveness of the reporting information, we first collected information related to cohorts from the manuscripts and then we searched Google Scholar and the manuscript citation lists for related publications and, if available, cohort websites that further detailed overall cohort characteristics. We did not use reference lists of publications to identify new cohorts, i.e., we did not conduct snowball sampling, but rather to gather more information on cohorts already included.

We extracted data on the study objectives, location of recruitment, dates of enrollment, funding sources, overall aim(s) stated by cohorts, types of exposures measured in women during or before pregnancy (e.g., demographic factors, infectious exposures, social exposures); pregnancy, infant, and child outcomes measured by the cohorts (e.g., biological, social, and cognitive measures); and types of maternal, infant, and child samples collected and made available by cohorts (e.g., sample type, frequency, storage, and accessibility). Where studies reported a range of follow-up times (e.g., 31–46 months of follow-up), we used the upper limit to calculate the mean follow-up time, because the upper limit reflected the intended follow-up period planned by the cohort, while the lower limit often represented dropouts or incomplete follow-ups. Lastly, we conducted a grey literature search for platforms or repositories that harmonise and disseminate or otherwise host participant-level data and study-level metadata across prepregnancy and pregnancy cohorts. We included platforms or registries if they provided access to individual participant-level data or metadata from multiple prepregnancy or pregnancy cohorts and were publicly accessible or described in peer-reviewed or grey literature sources. The results of the data charting process are presented in narrative form to identify patterns across cohorts in populations, structure, and the range of exposures, outcomes, biospecimens, and data-sharing practices examined in the review. As our objective was to summarize the landscape of existing pregnancy and prepregnancy cohorts, we did not review study quality. Since scoping reviews are intended to provide a broad overview of the scientific literature rather than evidence related to the findings of included studies, scoping reviews generally do not assess study quality [50, 54, 55].

## Results

**Fig. 1:**
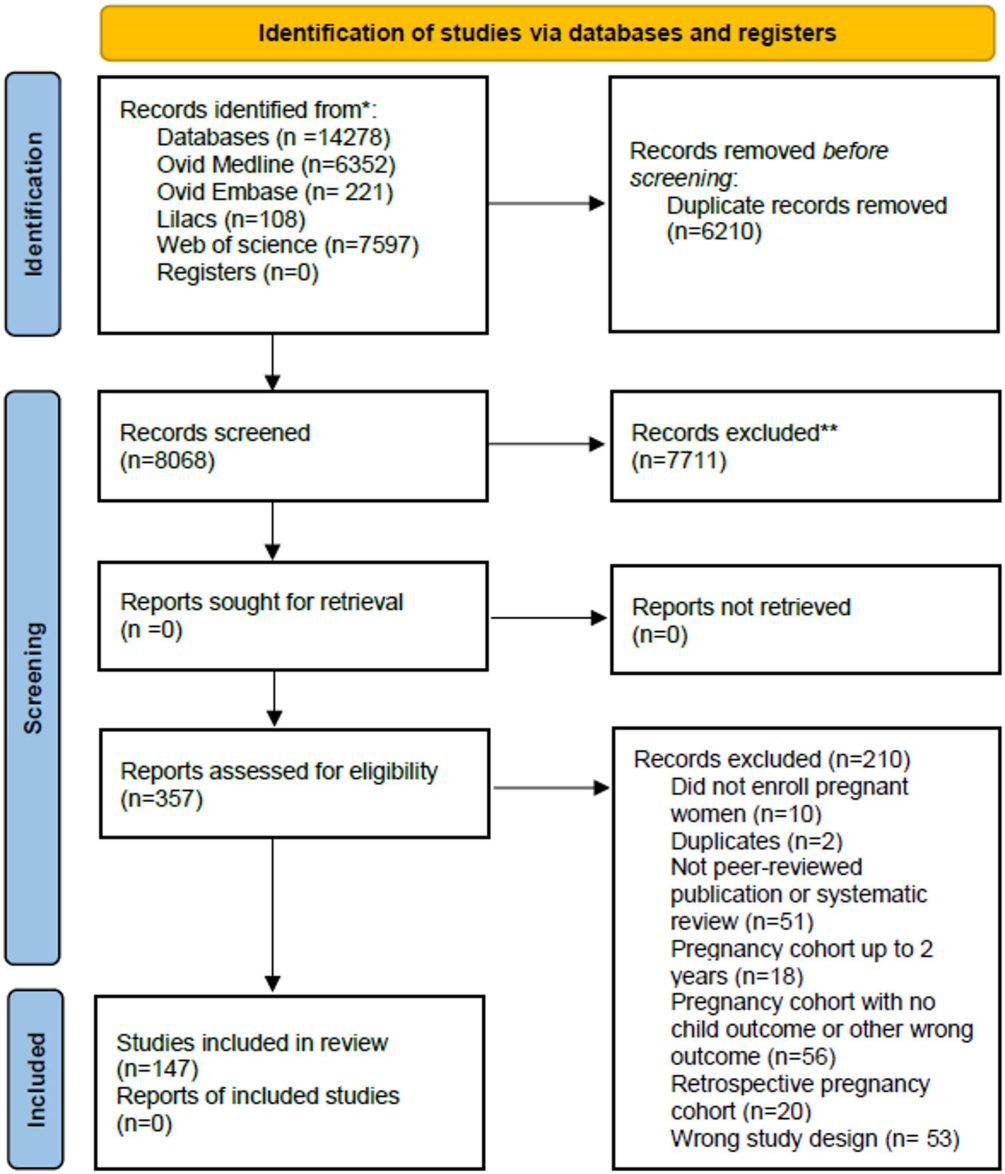
PRISMA flow diagram_REVISED

The systematic search results are presented in the Fig. 1 PRISMA flow diagram. After deduplication, we identified 8,068 citations that were screened for inclusion. We evaluated 357 full texts and extracted data from the 147 manuscripts that met our inclusion criteria. Studies were most commonly excluded due to wrong study design (e.g., reliance on national registries, electronic health records, RCTs, non-peer-reviewed publications, or systematic reviews), wrong patient population (e.g., cohorts not focused on pregnant women or women of reproductive age), wrong outcomes (e.g., studies not measuring birth or child outcomes), or pregnancy cohorts with fewer than two years of follow-up.

Table 1 provides an overview of the included pregnancy and prepregnancy cohorts. We identified three cohort profiles that provided general information about the cohorts, including overall aims, cohort setup, recruitment and eligibility criteria, types of data and samples collected, and study findings. The other 144 manuscripts were primary research articles investigating the effects of various prenatal exposures on child outcomes. These studies presented data from 56 cohorts in 26 countries. Two cohorts enrolled women before pregnancy [56, 57], and the other 54 enrolled pregnant women. We identified two pregnancy cohorts in Africa, four in Asia, five in Central and South America, seven in Oceania, 13 in North America, and 23 in Europe. One prepregnancy cohort was in Singapore [57], and the other recruited women in Mexico [56]. Five cohorts were limited to special populations, including farmworkers in California [58], Australian indigenous women [59], Australian women exposed to the 2011 Queensland floods [60], women with low household incomes [61], and adolescents aged 13 to 19 [62]. The number of participants enrolled in each cohort varied considerably, from 53 to 101,042 women (mean = 6,992) and 53 to 92,670 children (mean = 8,384). The Danish National Birth Cohort (DNBC) [63] and the Norwegian Mother and Child Cohort (MoBa) [64], which recruited over 95,000 women to participate from Denmark and Norway, respectively, were the largest cohorts. A few cohorts did not provide information on the number of women enrolled and children followed. When manuscripts, cohort profiles, or cohort websites did not explicitly state that the cohort was ongoing or that further follow-ups were planned, we considered them as having completed data collection.

**Table 1:** Ongoing and completed pregnancy and prepregnancy cohorts

**Cohort acronym**	**Cohort name**	**Cohort location**^a^	**Enrollment period**^a^	**Type of cohort**	**Number** **of** **women**	**Number** **of** **children**	**Duration of** **follow-up**^a^	**Number of** **follow-ups**^a^	**Ongoing** **or** **completed**^a^	**Source population (Community or health centre-based)**
ABCD	Amsterdam Born Children and Development Study	Netherlands (Amsterdam)	2003–2004	Pregnancy cohort	8,266	7,995	16	6	Ongoing	geographic region ONLY (not associated with a particular hospital or hospital system)
ACCESS	Asthma Coalition on Community, Environment and Social Stress project	US (Boston)	2002–2007	Pregnancy cohort	997	NS	6	14	Completed	other: prenatal service recipient + women attending Women, Infants and Children (WIC) programs
ALSPAC	Avon Longitudinal Study of Parents and Children	England (Bristol)	1991–1992	Pregnancy cohort	14,541	14,646	24	60	Ongoing	geographic region ONLY (not associated with a particular hospital or hospital system)c
AOF	All Our Families community-based cohort	Canada (Calgary)	2008–2010	Pregnancy cohort	3,200	3,200	8	6	Ongoing	geographic region ONLY (not associated with a particular hospital or hospital system)
APrON	Alberta Pregnancy Outcomes and Nutrition Study	Canada (Calgary, Edmonton)	2009–2012	Pregnancy cohort	2,189	2,189	12	7	Ongoing	NS
C-ABCS	China-Anhui Birth Cohort Study	China (Hefei, Ma’anshan, Wuhu, Jieshou, Lu’an, Ningguo)	2008–2010	Pregnancy cohort	14,027	13,454	15	6	Ongoing	geographic region ONLY (not associated with a particular hospital or hospital system)
C-MaMiE	Child outcomes in relation to MAternal Mental health in Ethiopia	Ethiopia (Butajira)	2005–2006	Pregnancy cohort	1,410	2,090	9	3	Ongoing	geographic region ONLY (not associated with a particular hospital or hospital system)
CANDLE	The Conditions Affecting Neurocognitive Development and Learning in Early Childhood Study	US (TN)	2006–2011	Pregnancy cohort	1,503	1,503	12	14	Completed	OTHER: THE first wave of recruitment was associated with a few clinics, and the second wave included community sources
CCREOH	Caribbean Consortium for Research in Environmental and Occupational Health Cohort Study	Suriname (Paramaribo, Nickerie)	2016–2019	Pregnancy cohort	1,143	12	4	3	Completed	the maternity ward of a hospital or group of hospitals (identified through where they give birth + geography)
CHAMACOS	Center for the Health Assessment of Mothers and Children of Salinas study	US, (CA)	1999–2000, 2010–2011	Pregnancy cohort	538	538	19	> 6	Ongoing	the maternity ward of a hospital or group of hospitals (identified through where they give birth + geography)
DNBC	The Danish National Birth Cohort	Denmark (Nationwide)	1995–2002	Pregnancy cohort	101,042	92,670	18	9	Ongoing	prenatal or antenatal service recipient + geography
EDEN	EDEN Mother–Child Cohort Study	France (Poitiers, Nancy)	2003–2006	Pregnancy cohort	1,899	1,899	18	11	Ongoing	the maternity ward of a hospital or group of hospitals (identified through where they give birth + geography)
EDMD	Early Determinants of Mammographic Density Study	US (California, Boston, Massachusetts and Providence, Rhode Island)	1959–1967	Pregnancy cohort	817	1,134	40	NS	Ongoing	prenatal or antenatal service recipient + geography
ELSPAC	The European Longitudinal Study of Pregnancy and Childhood	Czech Republic (Brno, Znojmo)	1991–1992	Pregnancy cohort	5,151	7,589	30	9	Ongoing	geographic region ONLY (not associated with a particular hospital or hospital system)
GIC	Gestational Iodine Cohort	Australia (Tasmania)	1999–2000	Pregnancy cohort	431	NS	15	4	Completed	prenatal or antenatal service recipient + geography
GUSTO	Growing Up in Singapore Towards healthy Outcomes	Singapore	2009- 2010	Pregnancy cohort	1,247	1,176	6	11	Completed	the maternity ward of a hospital or group of hospitals (identified through where they give birth + geography)
HDSS-León	The Nicaraguan Health and Demographic Surveillance System	Nicaragua (Leòn)	2002–2003	Pregnancy cohort	478	478	NS	NS	Completed	geographic region ONLY (not associated with a particular hospital or hospital system)
INMA	INfancia y Medio Ambiente—Environment and Childhood	Spain: Ribera d’Ebre, Menorca, Granada, Valencia, Sabadell, Asturias and Gipuzkoa (multiple cohorts)	1997–2008	Pregnancy cohort	3,944	3,768	13	8	Ongoing	geographic region ONLY (not associated with a particular hospital or hospital system)
KOMCHS	Kyushu Okinawa Maternal and Child Health Study	Japan (Kyushu Island, Okinawa Prefecture)	2007–2008	Pregnancy cohort	1,757	1,757	6	8	Completed	geographic region ONLY (not associated with a particular hospital or hospital system)
MoBa	The Norwegian Mother and Child Cohort Study	Norway (nationwide)	1999–2008	Pregnancy cohort	95,244	114,479	17	> 10	Ongoing	geographic region ONLY (not associated with a particular hospital or hospital system)
MUSP	The Mater-University of Queensland Study of Pregnancy	Australia (Brisbane)	1981–1983	Pregnancy cohort	6,753	7,223	30	6	Ongoing	the maternity ward of a hospital or group of hospitals (identified through where they give birth + geography)
MYPS	The Mothers’ and Young People’s Study (formerly known as Maternal Health Study)	Australia (Melbourne)	2003–2005	Pregnancy cohort	1,507	NS	10	6	Ongoing	the maternity ward of a hospital or group of hospitals (identified through where they give birth + geography)
NA	CHILD-SLEEP birth cohort	Finland (Tampere)	2011–2012	Pregnancy cohort	1,677	1,673	5	4	Completed	prenatal or antenatal service recipient + geography
NA	Generation R Study	Netherlands (Rotterdam)	2002–2006	Pregnancy cohort	9,778	9,749	16	11	Ongoing	geographic region ONLY (not associated with a particular hospital or hospital system)
NA	Gomeroi gaaynggal study	Australia (Tamworth, Walgett, New Castle)	2010–2014	Pregnancy cohort	227	220	5	5	Completed	prenatal or antenatal service recipient + geography
NA	Healthy habits for two (2005 follow-up)	Denmark (Aalborg, Odense)	1984–1987	Pregnancy cohort	11,980	11,144	30	2	Ongoing	geographic region ONLY (not associated with a particular hospital or hospital system)
NA	Project Viva Cohort	US (ME)	1999–2002	Pregnancy cohort	2,128	2,128	10	4	Ongoing	prenatal or antenatal service recipient + geography
NA	The Lifeways Cross-Generation Cohort Study	Ireland (West and East coast)	2002–2003	Pregnancy cohort	1,133	1,114	10	4	Ongoing	the maternity ward of a hospital or group of hospitals (identified through where they give birth + geography)
NA	The Mother–Child Cohort in Crete, Greece (Rhea Study)	Greece (Crete)	2007–2008	Pregnancy cohort	1,610	1,458	6	5	Completed	prenatal or antenatal service recipient + geography
NA	The Vitamin D in Pregnancy cohort study	Australia (Geelong)	2002–2005	Pregnancy cohort	400	400	11	4	Future follow-ups are subject to funding	hospital or group of hospitals catchment area (not linked to receipt of prenatal care or maternity care)
NA (1)	NA (1)	China (Hong Kong)	2005–2006	Pregnancy cohort	487	NS	3	1	Completed	the maternity ward of a hospital or group of hospitals (identified through where they give birth + geography)
NA (2)	NA (2)	England (Southampton)	1991–1992	Pregnancy cohort	596	NS	9	1	Completed	the maternity ward of a hospital or group of hospitals (identified through where they give birth + geography)
NA (3)	NA (3)	Italy (Rome)	"The project started about four years ago"—year not specified (publication minus 4 years = circa 2011)	Pregnancy cohort	53	53	3	3	Completed	hospital or group of hospitals catchment area (not linked to receipt of prenatal care or maternity care)
NA (4)	NA (4)	Canada (Vancouver)	2003–2009	Pregnancy cohort	118	118	6	1	Completed	geographic region ONLY (not associated with a particular hospital or hospital system)
NA (5)	NA (5)	US (Alabama)	1985–1988	Pregnancy cohort	740	NS	5	NS	Completed	prenatal or antenatal service recipient + geography
NA (6)	NA (6)	Finland (Tampere)	1989–1990	Pregnancy cohort	349	NS	17	6	Completed	prenatal or antenatal service recipient + geography
NA (7)	NA (7)	Mexico (Morelos)	2001–2006	Prepregnancy cohort	442	442	5	10	Completed	Other: prenuptial talks (before marriage, before pregnancy) and geographic location
NA (8)	NA (8)	Brazil (Sao Paulo)	2007–2007	Pregnancy cohort	120	120	4	10	Completed	Other: pregnant women who were included in a government program to monitor the prenatal period
NA (9)	NA (9)	Netherlands (exact location NS)	NS	Pregnancy cohort	174	174	6	6	Completed	NS
NA (10)	NA (10)	US (exact location NS)	NS	Pregnancy cohort	499	499	2	NS	NS	prenatal or antenatal service recipient + geography
NA (11)	NA (11)	Brazil (Pelotas)	2009–2011	Pregnancy cohort	828	670	3	2	Completed	prenatal or antenatal service recipient + geography
NCPP	National Collaborative Perinatal Project	US (nationwide)	1959–1966	Pregnancy cohort	48,197	54,390	7	8	Completed	prenatal or antenatal service recipient + geography
NEST	Newborn Epigenetics Study	US (Derham)	2005–2011	Pregnancy cohort	2,500	2,500	15	NS	Ongoing	prenatal or antenatal service recipient + geography
OCC	Odense Child Cohort	Denmark (Odense Municipality)	2010–2012	Pregnancy cohort	2,553	2,874	18	9	Ongoing	geographic region ONLY (not associated with a particular hospital or hospital system)
PEACHES	Programming of Enhanced Adiposity Risk in Childhood Early Screening	Germany (Bavaria, Düsseldorf, and northern Germany)	2010–2015	Pregnancy cohort	1,671	1,557	4	4	Completed	the maternity ward of a hospital or group of hospitals (identified through where they give birth + geography)
PELAGIE	Perturbateurs endocriniens, Étude Longitudinale sur les Anomalies de la Grossesse, l’Infertilité et l’Enfance	France (Brittany)	2002–2006	Pregnancy cohort	3,421	NS	19	4	Ongoing	NS
PROGRESS	Programming Research in Obesity, Growth, Environment, and Social Stressors Cohort	Mexico (Mexico City)	2007–2011	Pregnancy cohort	1,054	948	12	4	Ongoing	prenatal or antenatal service recipient + geography
QF2011	Queensland Flood study	Australia (Queensland)	2011–2012	Pregnancy cohort	230	NS	6	6	Completed	geographic region ONLY (not associated with a certain hospital or hospital system)
Raine	The Western Australian Pregnancy Cohort Study	Western Australia	1989–1991	Pregnancy cohort	2,900	2,868	30	15	Ongoing	prenatal or antenatal service recipient + geography
RPGEH Pregnancy Cohort	Research Program on Genes, Environment and Health Pregnancy Cohort (pregnancy cohort is a part of the more extensive study RPGEH)	US (California)	2010–2014	Pregnancy cohort	16,977	NS	NS	NS	Completed	Other: recipient of KPNC (Kaiser Permanente Northern California) healthcare service
SCDS	The Seychelles Child Development Study	Seychelles	1989–1990	Pregnancy cohort	779	779	30	10	Ongoing	NS
SEATON	Study of Eczema and Asthma To Observe effect of Nutrition	Scotland (Aberdeen)	1997–1999	Pregnancy cohort	2,000	1,924	15	5	Ongoing	prenatal or antenatal service recipient + geography
SGA	The Scandinavian Small-for-Gestational Age pregnancy and birth cohort	Norway and Sweden (Bergen, Uppsala, Trondheim)	1986–1988	Pregnancy cohort	NS	NS	26	7	Completed	geographic region ONLY (not associated with a particular hospital or hospital system)
SLCDS	South London Child Development Study	England (London)	1986	Pregnancy cohort	252	252	25	6	Completed	prenatal or antenatal service recipient + geography
SMOK	SSRI in pregnant mothers, the outcome of the kids'study	Northern Netherlands	2007–2010	Pregnancy cohort	111	111	2.5	1	Completed	Other: women were recruited via newspapers, midwives, general practitioners, gynaecologists, and psychiatrists (= geography is limited to the vicinity of two Level-2 hospitals in northern Norway, but the source of the population is not connected to the hospitals specifically but rather living area, i.e. pregnant women living in the proximity of mentioned hospitals)
S-PRESTO	Singapore Preconception Study of Long-Term Maternal and Child Outcomes	Singapore	2015–2017	Prepregnancy cohort	475	373	4	11	Ongoing	Other: preconception clinic established to recruit women and public recruitment

^a^Country (city) (where available)

^a^Year enrollment started and finished

^a^Child age in years at last reported follow-up

^a^Number of reported follow-ups of child during follow-up period

^a^Considered completed unless explicitly stated otherwise or mentioned future follow-up

Because the focus of this review was on the design and focus of prepregnancy and pregnancy cohorts, we did not collect data on how many participants in each cohort did not complete follow-up or missed certain time points. Cohorts that had stopped collecting data followed infants and children between 36 months and 26 years of age (mean = 7.3 years).

The number of follow-ups ranged from one to over 60 (mean = 6.3). We identified 28 cohorts that were ongoing at the time of data extraction. Of those, children were followed between 4 years of age and adulthood, including several cohorts that followed children from more than 30 years. The DNBC [63] recruited its first participant in 1995, and the MoBa cohort [64] in 1999. Both these cohorts were ongoing at the time of data extraction, having followed children into young adulthood. The Mater-University of Queensland Study of Pregnancy (MUSP) [65], the Healthy Habits for Two cohorts [66], the Western Australian Pregnancy Cohort Study (Raine) [67], the Seychelles Child Development Study [68], the Early Determinant of Mammographic Density Study [69], and the European Longitudinal Study of Pregnancy and Childhood (ELSPAC) [70] were the longest-running cohorts with a follow-up period of over 30 years each. The frequency of follow-ups varied greatly. Generally, infants were followed up multiple times yearly in their first one to three years. Following infancy, the frequency of follow-up ranged from annually to once every five years. We could not assess the follow-up schedule for children over 18 in the Healthy Habits for Two cohort [66].

**Fig. 2:**
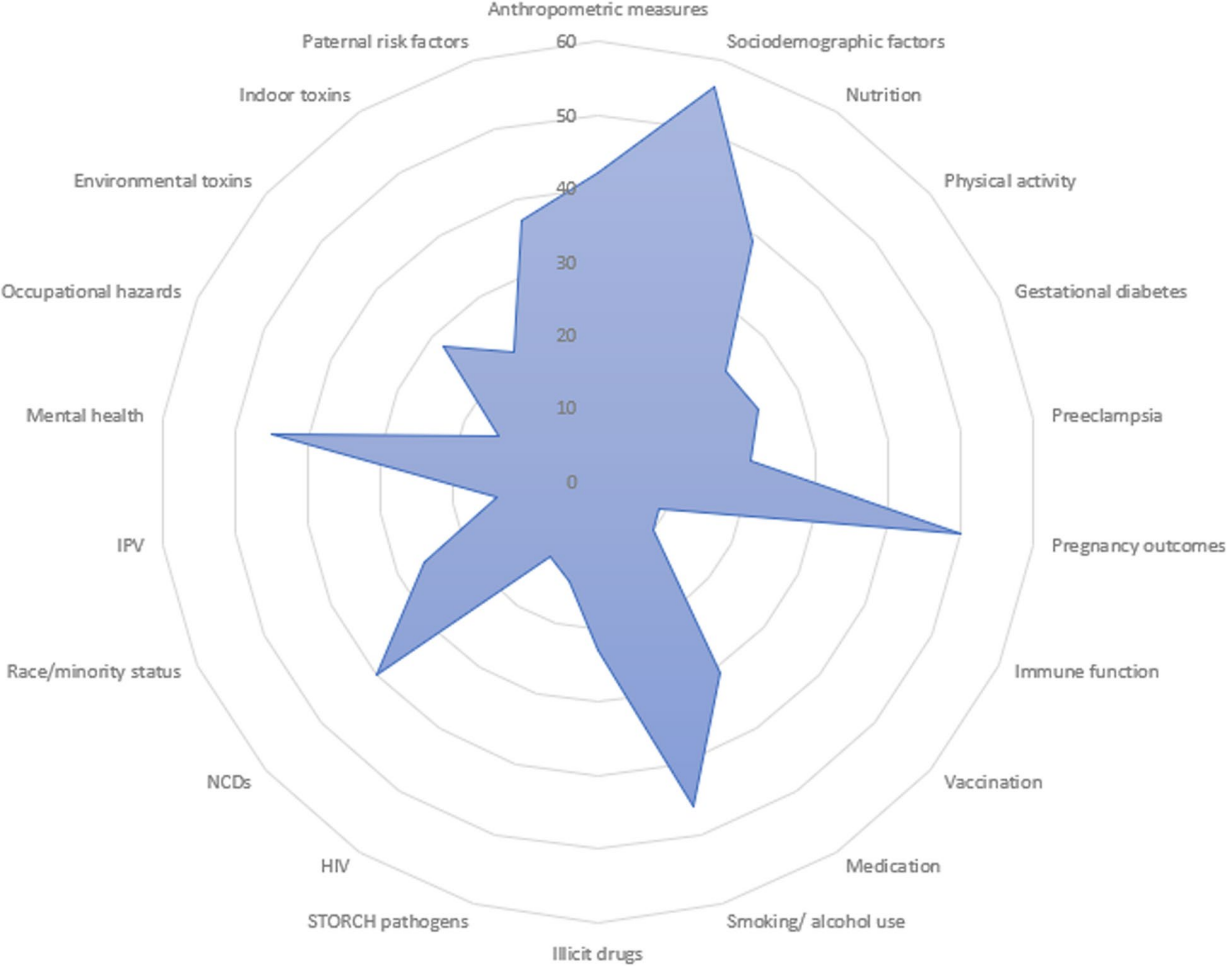
Spider plot of the number of cohorts that measured given perinatal risk factors

**Fig. 3:**
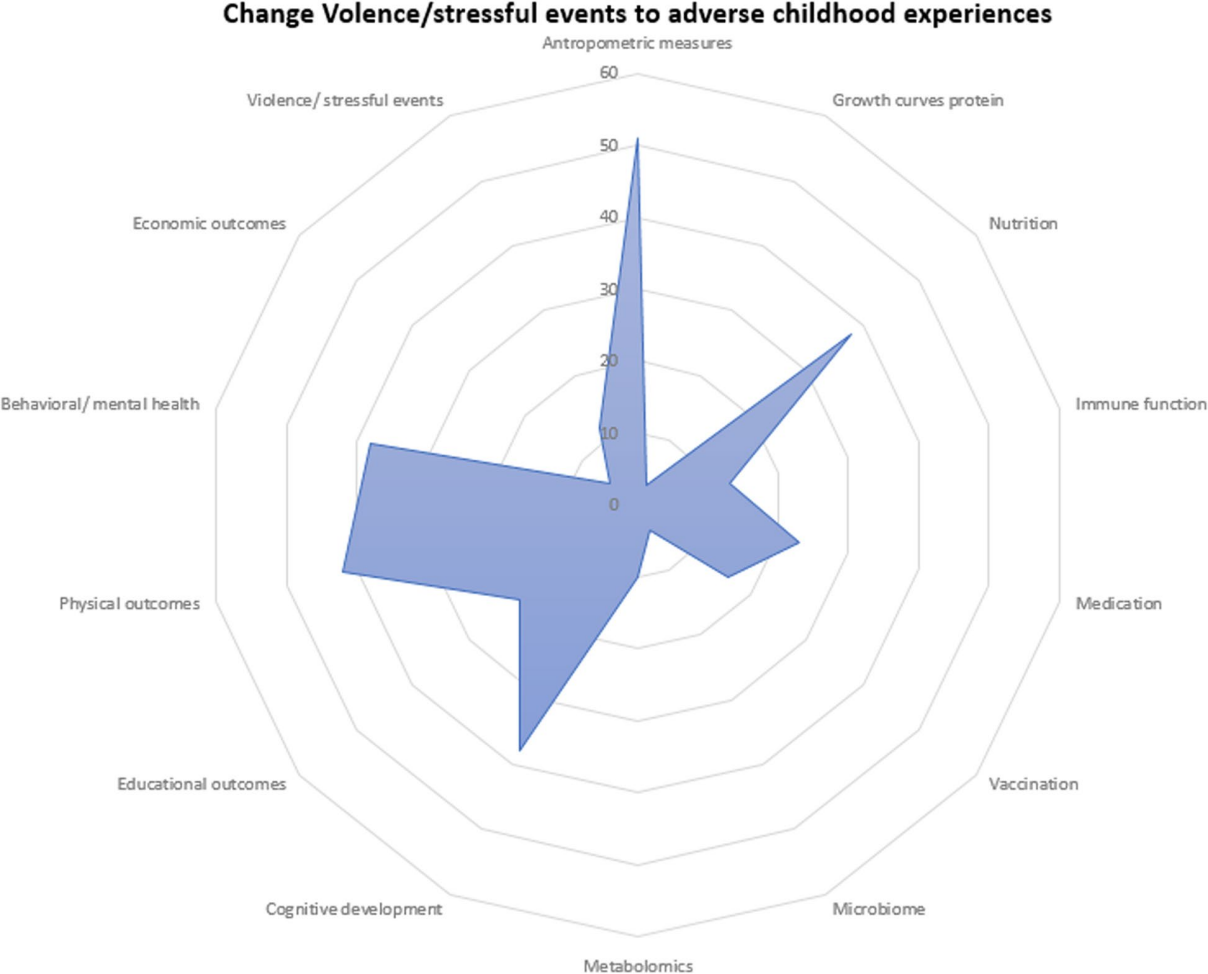
Spider plot of the number of cohorts that measured given child outcomes

Cohorts generally had broadly defined aims and explored multiple prenatal exposures and fetal, infant, and child outcomes. Most cohorts with more focused aims assessed the relationship between maternal nutrition and child cognitive development, maternal environmental exposures and child development [58, 71, 72], maternal psychological disorders and child cognitive outcomes [73], maternal racial and ethnic differences and child health, and the effects of intergenerational trauma [74] and stress related to environmental disasters [60]. Additional File 5 presents cohort aims, and Additional File 6 provides an overview of the types of maternal exposures measured across pregnancy and prepregnancy cohorts. The spider plots in Figs. 2 and 3 show how many pregnancy and prepregnancy cohorts measured different categories of maternal exposures and child outcomes. Most cohorts collected data on maternal anthropometric measures, sociodemographic factors, nutrition, and pregnancy outcomes, while relatively few assessed STORCH (Syphilis, Toxoplasmosis, Other (Hepatitis B, HIV, Rubella, Cytomegalovirus, and Herpes Simplex Virus)) pathogens or IPV (Fig. 2).

Child anthropometry, cognitive development, and behavioral or mental health outcomes were the most frequently measured child outcomes (Fig. 3). Fewer cohorts examined adverse childhood experiences, microbiome composition, or metabolomic profiles.

### Nutrition exposures

Thirty-seven of the 56 pregnancy and prepregnancy cohorts (66%) collected data on maternal nutrition during and or after pregnancy. Forty-four individual studies investigated the effects of nutrition-related exposures, and several additional studies included maternal nutrition as a confounder. Eight studies assessed general dietary patterns, overall diet quality, and maternal nutritional status. Additional studies examined prenatal exposure to specific diets (e.g., Mediterranean, gluten-free), nutritional restrictions, or certain foods or drinks (e.g., seafood, caffeine, sweetened beverages). Prenatal intake or deficiency of vitamins and minerals was assessed by 18 studies, which examined maternal vitamin D (*n* = 10), B12 (*n* = 1), E (*n* = 1), calcium (*n* = 1), iron (*n* = 2), and iodine intake (*n* = 7). Studies examined the effect of maternal nutritional exposures on child’s physical health (*n* = 15), cognitive or neurodevelopmental (*n* = 16), behavioural and mental health (*n* = 5), anthropometric (*n* = 10), and educational (*n* = 1) outcomes.

### Anthropometric measures & non-communicable diseases

Anthropometric measures such as pregpregnancy or maternal BMI, or weight gain during pregnancy were recorded in 42 of the 56 cohorts (75%). Twenty-four cohorts (42%) collected information on maternal exposure to NCDs. Potential participants with any underlying medical conditions, a history of chronic disease, or reporting current physical health issues were excluded from five cohorts. Eight studies included NCDs as the primary exposure. Five of these studies assessed the effects of prenatal thyroid dysfunction on child attention deficit hyperactivity disorder (ADHD) [75], depression and anxiety [76], educational attainment [77], and brain morphology and IQ [78, 79]. One study focused on maternal risk for type 2 diabetes mellitus (T2DM) and child’s cognitive abilities [80]. Another assessed the relationship between late-pregnancy dysglycemia in obese pregnant women and childhood weight gain [81]. One study investigated maternal obesity before pregnancy and child cognitive development [82], and one study examined the effects of maternal preeclampsia and gestational hypertension on childhood and adolescent behavioural problems [7].

### Infectious disease and markers of immune function

Nine cohorts (16%) recorded maternal exposure to STORCH pathogens. Six additional cohorts collected medical records or general health information without stating whether those included ID exposures. One study with the Western Australian Pregnancy Cohort data assessed the relationship between prenatal exposure to herpes, rubella, hepatitis B, or rheumatic fever and child-adolescent eating disorders [83]. No other studies examined the relation between ID exposures and child or later life outcomes. Nine cohorts (16%) collected biomarkers related to maternal immune function, including C-reactive protein and T-cell count. Two studies reported on maternal biomarkers and child outcomes. One study from the Rhea Pregnancy Cohort looked at the effect of maternal C-reactive protein on child adiposity [84]. Another study from the Gomeroi Gaaynggal cohort assessed the relationship between maternal T-cell count and child growth, renal disease, and diabetes [59]. We did not identify other pregnancy or prepregnancy cohort studies focusing on the relationship between immune function and child outcomes.

### Medication & vaccine exposures

Thirty cohorts (54%) captured data on maternal medication use. Eight studies assessed medication use during pregnancy as the primary exposure. These included investigating the effect of antibiotic use on childhood overweight [85] and diabetes [86], antiepileptic drugs and language development [87], selective serotonin reuptake inhibitors (SSRIs), and language and neurodevelopment [88–91], thyroid medication and language and motor development [92]. Ten cohorts recorded maternal vaccination status, one of which collected data on COVID-19 vaccination only [93]. We did not identify any cohort studies that aimed to investigate the effects of vaccine exposure during pregnancy.

### Environmental exposures

We assessed whether cohorts collected any data on environmental toxins, categorizing them as indoor or outdoor exposures and recording which specific toxins were investigated. As relatively few studies (n = 12) focused on environmental exposures, no patterns emerged regarding specific toxins studied, and the outcomes examined varied widely.

Overall, twenty-eight cohorts (50%) captured exposure to outdoor environmental toxins. Seven studies examined prenatal exposures, including mercury [94, 95], lead [96], long-chain polyunsaturated fatty acids (LCPUFA) [97], polychlorinated biphenyl (PCB) [98], synthetic insecticides [99, 100], gasoline exhaust [101], chemicals used to treat drinking water [102], and any toxin [71]. These studies assessed a range of child health outcomes, including cognitive development [71, 94], neurodevelopment [95, 102], growth [96], leukaemia [101], behaviour [100], and eating disorders [83]. Indoor environmental toxins were measured in 14 (25%) cohorts, with five studies examining the effects of indoor toxins specifically. These included maternal exposure to flame retardants and disinfection by-products in relation to cognitive outcomes [102, 103], per- and poly-fluoroalkyl substances (PFASs) and childhood asthma, allergies, and common IDS [104], phthalates and bisphenols in relation to childhood blood pressure [105], and pyrethroid and chlorpyrifos and child ADHD [99].

### Demographic and social exposures

All cohorts recorded socio-demographic factors, including maternal age, educational status, parity, relationship status, personal or household income, and employment status. All studies included several demographic and social measures as control variables. A smaller subset of studies recorded occupation, household size, and country of birth. Twenty-six cohorts (46%) recorded maternal race or ethnicity.

### Violence or traumatic events

Eleven cohorts collected IPV data (20%), and three studies assessed IPV as the primary exposure. These examined the effects on the risk of child abuse and neglect [106], child language development [107], and physical growth [108]. No studies focused on maternal exposure to violence other than IPV. One study focused on the effects of flood-related PTSD and prenatal maternal stress in Australian mothers on child school-age anxiety [60]. The effects of maternal mental health, including depression and anxiety, general psychosocial factors, the experience of traumatic life events, and exposure to stress, were explored in 32 studies. One study measured life events during pregnancy typically perceived as stressful and their effects on child language abilities [109].

### Child outcomes

Additional File 7 details the types of infant and child outcomes measured across pregnancy and prepregnancy cohorts. Fifty-one (91%) cohorts collected infant or child anthropometric data. Most cohorts also collected data on childhood NCDs (e.g., asthma, eczema, allergies) or other physical outcomes (*n* = 42), child nutrition (*n* = 38), cognitive development (*n* = 38), and behavioural or mental health outcomes, including infant sleeping patterns, crying, depression, eating disorders, and externalising or internalising behaviour (*n* = 38). The least common outcomes measured by cohorts included growth curves (*n* = 3), microbiome (*n* = 4), economic outcomes (*n* = 5), the experience of violence and stressful events (*n* = 12), metabolomics (*n* = 9), and immune function (*n* = 9). Fourteen (25%) and 17 (30%) cohorts recorded child vaccination status and medication use, respectively. We identified 64 studies that focused on the effects of various prenatal exposures on children’s cognitive development, behavioural outcomes, or mental health. Thirty-five studies measured physical outcomes in children, including blood pressure, bone mineral content or bone mass, and NCDs, including diabetes, allergies, asthma, and leukaemia, among others. Sixteen studies examined anthropometric measures as the primary child outcome, including general growth, skeletal growth, weight gain, and childhood obesity or overweight. Educational outcomes and experience of violence and stress were assessed by four and two studies, respectively.

### Sample collection and availability

Cohort sample collection is detailed in Additional File 8. One of the two prepregnancy cohorts did not collect any samples [56]; the other collected maternal samples before and during pregnancy and fetal samples at birth [12]. Thirty-six of the 56 pregnancy and prepregnancy cohorts (64%) collected at least one type of sample during pregnancy, the most common ones being blood (*n* = 33), urine (n = 16), saliva (*n* = 8), and hair (*n* = 7). The timing and frequency of sample collection during pregnancy varied across cohorts. Most studies collected samples twice during pregnancy. Some studies focused on specific trimesters, with the first and third being commonly targeted. A detailed description of the timing and frequency of sample collection can be found in Additional File 8. Eight cohorts collected paternal samples during or following pregnancy. Twenty-seven cohorts (48%) included fetal samples, including cord blood or placental tissue, usually taken at birth. Sixteen and 21 cohorts collected infant and child samples, respectively. Most publications did not indicate whether samples were available to outside researchers. Through additional research, we were able to identify biobanks or biorepositories and sample access procedures for the MoBa [110], Avon Longitudinal Study of Parents And Children (ALSPAC) [93], Scandinavian Successive Small for Gestational Age (SGA) [111], and Center for the Health Assessment of Mothers and Children of Salinas (CHAMACOS) [112] cohorts.

### Platforms that host data from pregnancy and prepregnancy cohorts

Whether data had been shared to a registry or platform to facilitate its reuse could not be determined from most publications. Where data-sharing policies were mentioned in publications, studies said data could be made available upon reasonable request to the authors [71, 90, 113, 114]. Through reviewing study websites, we were able to identify data access procedures for 7 of the 56 cohorts, including the ALSPAC [93], Conditions Affecting Neurocognitive Development and Learning in Early Childhood (CANDLE) [115], CHAMACOS [112], DNBC [63], MoBa [110], SGA [111], and the Vitamin D in Pregnancy Cohort [116].

## Discussion

In this comprehensive scoping review, we extracted data from 127 studies representing 54 pregnancy and two prepregnancy cohorts that followed infants and children beyond two years of age. Anthropometric measures, nutrition, and NCDs were the most commonly measured maternal exposures. Over half of included studies investigated child anthropometric, cognitive development, behaviour, and mental health outcomes. Half of the cohorts were based in Europe. While Africa and Asia host most of the world’s population, only 6 pregnancy cohorts were located there.

What we don’t know can be as informative as what we know. While many studies examined the relationship between dietary and vitamin exposures during pregnancy and later life outcomes, only a few cohorts recorded maternal exposure to ID and child outcomes and only one study considered ID as the primary maternal exposure. The lack of focus on ID exposures during pregnancy may be because of difficulties in measuring incident infections or because IDs are a lesser concern for the high-income countries (HICs) where most of the cohorts were based. Infections during pregnancy may be asymptomatic or present with nonspecific symptoms, making clinical detection difficult [117]. Additionally, standard diagnostic tools may lack the sensitivity or specificity required to ascertain the timing of infection relative to gestational age, which significantly influences outcomes, further complicating ID ascertainment in pregnancy [118]. In HICs, the prevalence and impact of ID during pregnancy are generally lower due to better healthcare infrastructure, widespread immunization programs, and improved sanitation [119, 120]. Consequently, research priorities in these regions may focus on NCDs, leading to an underrepresentation of ID exposures in pregnancy cohorts based in HICs. That said, the recent rise in infant deaths related to congential syphilis in Queensland, Australia highlights the ongoing relevance of studying IDs during pregnancy, even in HICs [121]. The need for improved data on maternal infectious exposures is particularly evident during public health emergencies. The 2015–17 ZIKV epidemic highlighted the importance of pregnancy and prepregnancy cohorts in the context of emerging infectious diseases [122]. In light of this, our findings emphasize the importance of enhancing exposure measurement practices within cohorts.

The stage and intensity of infection and timing of treatment initiation are strong predictors of pregnancy outcomes and likely related to the incidence and spectrum of later-life developmental effects, therefore, pregnancy cohorts should aim to record and systematically monitor ID exposures [123, 124]. A systematic review of maternal syphilis and pregnancy outcomes found that studies of infectious exposures in pregnant women had limited follow-up time, were restricted to symptomatic pregnant women, and were subject to misclassification and selection bias when infection was not captured by serology because of test sensitivity or when infection occurred following the study visit [125]. Studies of STORCH pathogens generally focus on pregnancy outcomes rather than longer-term effects [32, 125]. Many STORCH studies do not consider the possibility of co-infection [126], and some STORCH infections are not included in routine pregnancy screening [127, 128].

The lack of systematic exposure assessment was also evident in studies examining medications and vaccinations during pregnancy. Similar to ID exposures, only a few included studies assessed exposure to medicines during pregnancy. These studies evaluated the relation between maternal antibiotics, antiepileptic drugs, and SSRI use and child outcomes. While a few studies asked about vaccine histories, we did not identify any studies of vaccine exposures during pregnancy. Systematic reviews of studies of vaccine exposures during pregnancy found that existing studies were limited and focused on immediate infant rather than longer-term child outcomes [113, 129–133]. This limited scope leaves significant uncertainty regarding the developmental and health impacts of prenatal vaccine exposure beyond the neonatal period.

This gap in exposure data is compounded by the broader issue of pregnant women being underrepresented in safety and efficacy studies. They are generally excluded from vaccine and medication safety and efficacy studies, making it difficult for pregnant women and providers to make informed decisions about vaccination or medication use during pregnancy. Pregnancy cohorts typically lack information on drug and medication exposures, and pregnancy registry data lacks information on medication exposures [134]. Selection bias, small sample size, retrospective and self-reported data collection, and inadequate information on timing and dosage complicate the assessment of medication exposures during pregnancy [134]. Most pharmacovigilance studies that include pregnant women are cross-sectional [135], precluding causality assessment. Cohort data on maternal medication and vaccine exposures can facilitate post-marketing assessment of the risks and benefits of maternal medication exposure and longer-term child outcomes.

Other critical but underexamined exposures include those related to climate and the environment. Although climate-related exposures, including extreme heat, wildfire smoke, and flooding, are increasingly recognized as significant threats to MCH, we did not identify any pregnancy or prepregnancy cohorts that measured these exposures. Studies on the relationship between environmental or workplace exposures and child outcomes were also limited, given the number of potential exposures of concern. This absence may reflect historical underprioritization of climate-health linkages in maternal and child research or methodological challenges in linking geospatial environmental data to individual-level pregnancy records. Climate-related exposures often vary by region and season, requiring granular data and long-term follow-up to detect meaningful associations. Addressing these gaps will require the integration of environmental monitoring and cohort data, investment in climate-sensitive exposure assessment tools, and prioritization of MCH research that relates to vulnerable populations in regions experiencing disproportionate climate-related health burdens.

Other important social determinants of health were also sparsely assessed. While 11 cohorts collected data on IPV, only three studies included IPV as the exposure of interest. Fewer than half of the cohorts measured race, an important marker of child health outcomes.

Biological sample collection varied across cohorts, with notable gaps in follow-up. Over 60% of cohorts collected samples during pregnancy, and about half collected fetal samples. Fewer than half collected infant or child samples. Blood was the most commonly collected sample type. Most publications did not describe how to access pregnancy or prepregnancy cohort data or samples, although we were able to identify access procedures for four cohorts through their websites. We only identified two prepregnancy cohorts, representing a missed opportunity to understand how prior exposures affect pregnancy, fetal, infant, and child outcomes. The lack of guidance on how to access pregnancy and prepregnancy cohort data and biospecimens may reflect a range of structural, ethical, and logistical barriers. Data and sample sharing require clearly specified, well-structured governance procedures, broad consent for future use from research subjects, data and sample use agreements, and material transfer agreements, which may deter investigators from formalizing access procedures or sharing at all. Additionally, concerns over participant privacy, especially for sensitive exposures and biospecimens, may limit transparency. In some cases, cohorts may lack the infrastructure, funding, or personnel needed to support data and sample sharing, particularly if the original studies were not designed with long-term access or interoperability in mind. Addressing these barriers will require investments in data governance frameworks that support secure and equitable sharing, clear communication of access procedures, harmonization of cohort metadata to facilitate discoverability, and support to address concerns around broad consent for future use.

Improving data accessibility would be essential to unlocking the full potential of existing cohorts. We were not able to identify how data could be accessed for most cohorts from the cohort publications. Further work could explore how cohort metadata catalogues, e.g., Cohort and Longitudinal Studies Enhancement Resources (CLOSER), the European Open Science Cloud (EOSC), or other cohort metadata catalogues, could be used to identify cohorts that collect specific exposure or outcome data and how those data can be accessed.

### Strengths & limitations

Our scoping review had several important strengths. The comprehensive information on the landscape of pregnancy and prenatal cohorts that follow infants to at least two years of age can help researchers and funders understand the focus of existing studies and identify gaps in coverage of maternal exposures and associated child outcomes. We did not limit our search by geography or language of publication. We extracted data from included publications and looked for additional information on the identified cohorts from related websites and other publicly available documentation. The review has some limitations. While we aimed to comprehensively capture existing pregnancy and prepregnancy cohorts that followed infants to at least two years, some may have been missed despite our extensive search strategy. Because the search strategy targeted publications rather than cohort websites, cohort publications that did not meet the inclusion criteria were excluded even when the cohort itself could have been included if the inclusion criteria had been applied to the cohort. We did not search the websites of all cohorts screened in the title-abstract or full text phases, so we could have missed including pregnancy or pregnancy cohorts that reported a study population recruited at birth, even though the larger initiative also included women recruited during pregnancy. Additionally, we relied on available publications, cohort websites, and other cohort-related documentation to comprehensively describe exposures and outcomes measured, meaning that data collected within cohorts that were not explicitly reported in the publication or associated documentation would not have been reported here. Cohort sizes might be overestimated, as we recorded the number of recruited participants with at least one follow-up, but did not take into consideration dropouts or missed follow-up visits. Similarly, while we categorized follow-up frequency of infants and children (e.g., more frequent in the first three years of life), we did not systematically document the exact ages of follow-up assessments. Lastly, since we chose to focus this review on pregnancy and prepregnancy cohorts that followed infants to at least two years of age, we did not describe the many thousands of pregnancy and prepregnancy cohorts that describe fetal and infant outcomes from birth until before two years of age.

### Policy recommendations

Table 2 provides a summary of review findings as they relate to different maternal exposures, including recommendations for future research investments. Only a few included cohorts assessed maternal exposure to medications, antibiotics, antiepileptic drugs, or SSRIs and even fewer asked about vaccine histories. No studies evaluated vaccine exposures during pregnancy as a primary focus. Existing studies of maternal vaccine and medication exposures are typically limited by small sample sizes, selection bias, retrospective and self-reported data collection, and inadequate information on timing and dosage. Most pharmacovigilance studies involving pregnant women are cross-sectional, which restricts the ability to assess causality. Moreover, pregnancy cohorts rarely include standardized, prospective monitoring of medication or vaccine exposures, and registry data often lack detail on long-term child outcomes.

**Table 2:** Overview of maternal exposures and recommendations for future research

Exposure Category	Well Studied?	Barriers to Measurement	Future Research Needs
Nutrition and diet	Yes	Commonly included in baseline data	Greater granularity of nutrient subtypes & timing
Anthropometry	Yes	Routinely collected at antenatal visits	Linkage with long-term growth trajectories
Sociodemographic factors	Yes	Readily available from self-report or registry data	Intersectional analysis with health equity focus
Race and ethnicity	Partially	Varies by context, often inconsistently categorized	Systematic inclusion & standardized coding
Mental health	Yes	Validated tools exist for self-report	Integration with biomarker or epigenetic data
Interpersonal violence & IPV	Partially	Stigma, underreporting, sensitive nature	Validated tools for sensitive data, trauma-informed protocols
STORCH pathogens	No	Diagnostic accuracy related to presence of symptoms, generally requires clinical diagnosis but may be asymptomatic, low prevalence in HICs, co-infection complexity	Routine screening & integration with surveillance data; Expanded serologic testing, accounting for multiple pathogens
Medications & vaccines	No	Lack of inclusion in cohort protocols, ethical concerns, recall bias	Standardized reporting, inclusion in cohort design, linkage with pharmacovigilance
Environmental or workplace exposures	No	Diverse exposure sources, lack of standard metrics	Improved exposure assessment tools, harmonized definitions
Climate-related exposures	No	Not routinely monitored in pregnancy cohorts, region-specific data needs	Environmental monitoring linkage, regional cohort representation
Substance use	Partially	Social desirability bias, underreporting, variable definitions	Multimodal assessment (biological + survey), longitudinal tracking

HICs = High-income countries; IDs = infectious diseases; IPV = Intimate partner violence; STORCH = Syphilis, Toxoplasmosis, Other (Hepatitis B, HIV, Rubella, Cytomegalovirus, and Herpes Simplex Virus

To address these limitations, future pregnancy and prepregnancy cohorts should incorporate structured data collection tools to capture medication, vaccine, and infectious disease exposures with precise timing and dosage information, ideally through linkage with electronic health records or prescription registries. Systematic screening for STORCH pathogens, including co-infections, and inclusion of both symptomatic and asymptomatic infections would improve the quality of maternal ID exposure ascertainment. Longer term follow-up, combined with harmonized exposure definitions and integration with pharmacovigilance systems, would support more robust assessment of the relation between medication and vaccine exposures and long-term developmental outcomes and enhance the utility of cohorts for informing clinical and regulatory decision-making.

To improve coordination and data harmonization across cohorts, stakeholders could prioritize the development and adoption of common data elements, use of shared ontologies, and metadata catalogues to enable efficient discovery and cross-study synthesis of variables for pooled analyses. For example, researchers could leverage cohorts identified in this review to conduct pooled analyses on the impact of prenatal exposures (e.g., environmental toxins, maternal mental health) on adolescent mental health outcomes using harmonized variables. Initiatives such as the Maelstrom Research Metadata Catalogue [136] and the Global Pregnancy CoLab [137] can serve as platforms to promote interoperability and data reuse.

Key research priorities emerging from this review include identifying context-specific and common maternal exposures influencing long-term child development, examining intergenerational effects of preconception exposures, and addressing how social determinants and maternal ID and environmental exposures influence developmental trajectories.

## Conclusions

Prepregnancy and pregnancy exposures are central to understanding the distribution of birth and later-life developmental outcomes. The most commonly described maternal exposures for pregnancy and prepregnancy cohorts were nutrition, NCDs, and demographic factors. Children’s mental, behavioural, neurodevelopmental, and physical outcomes were the most commonly measured outcomes. Maternal ID, medication, climate, environmental, and vaccine exposures and infant or child outcomes like microbiome, immune function, and economic impacts were underrepresented. While core perinatal exposures such as nutrition, anthropometry, and sociodemographic factors were commonly measured, critical exposures with implications for public health and health equity, including IDs, medication and vaccine use, environmental hazards, and climate-related exposures, were substantially underrepresented in pregnancy and prepregnancy cohorts. To support meaningful causal inference and advance MCH research, future cohorts should prioritize systematic measurement of these exposures, integrate longitudinal follow-up beyond infancy, and establish transparent data and sample access protocols.

Coordination between pregnancy and prepregnancy cohorts can facilitate cross-population inference and build a better understanding of how maternal exposures shape later-life cognitive, behavioural, and developmental outcomes. Our findings can help researchers understand what resources exist to explore the relation between maternal exposures and child, adolescent, or adult offspring outcomes. This mapping exercise can also inform efforts to reuse participant-level data across ongoing or completed pregnancy and prepregnancy cohorts and support the development of a research agenda to set priorities for future investments to understand the proximal causes of short and long-term adverse developmental outcomes or chronic conditions.

## Supplementary Information

Supplementary Material 1.

## Data Availability

The author confirms that all data generated or analysed during this study are included in this published article and its supplementary information files.

## Abbreviations

ADHD: Attention deficit hyperactivity disorder
ALSPAC: Avon Longitudinal Study of Parents And Children
BMI: Body-mass index
CANDLE: Conditions Affecting Neurocognitive Development and Learning in Early Childhood
CHAMACOS: Center for the Health Assessment of Mothers and Children of Salinas
CHD: Congenital Heart Disease
CMV: Cytomegalovirus
COVID-19: Coronavirus Disease 2019
CVD: Cardiovascular Disease
DNBC: Danish National Birth Cohort
ELSPAC: European Longitudinal Study of Pregnancy and Childhood
HICs: High-income countries
HSV: Herpes simplex virus
ID: Infectious disease
IPV: Intimate Partner Violence
LBW: Low birth weight
LCPUFA: Long-chain polyunsaturated fatty acids
MCH: Maternal and child health
MeSH: Medical Subject Headings
NCD: Non-communicable disease
PCB: Polychlorinated biphenyl
PFASs: Per- and Poly-Fluoralkyl Substances
PRISMA: Preferred Reporting Items for Systematic Reviews and Meta-Analyses
PRISMA-ScR: PRISMA Extension for Scoping Reviews
PTSD: Post-Traumatic Stress Disorder
RCTs: Randomized Controlled Trials
SGA: Small for Gestational Age
SSRIs: Selective serotonin reuptake inhibitors
STORCH: Syphilis, Toxoplasmosis, Other (Hepatitis B, HIV, Rubella, Cytomegalovirus, and Herpes Simplex Virus)
T2DM: Type 2 diabetes mellitus
ZIKV: Zika virus
