# Supplementary material for: The landscape of pregnancy and prepregnancy cohorts: a scoping review
Source: BMC Pregnancy Childbirth. 2025 Oct 28;25:1146. doi: 10.1186/s12884-025-07949-7 (PMC12570564; doi:10.1186/s12884-025-07949-7)
Supplement: Supplementary file 1 — Supplementary Material 1. [file 12884_2025_7949_MOESM1_ESM.pdf]

Supplementary information

Additional File 1: Preferred Reporting Items for Systematic reviews and Meta-Analyses extension for Scoping Reviews (PRISMA-ScR) Checklist

SECTION	ITEM	PRISMA-ScR CHECKLIST ITEM	REPORTED ON PAGE #
**TITLE**			
Title	1	Identify the report as a scoping review.	1
**ABSTRACT**			
Structured summary	2	Provide a structured summary that includes (as applicable): background, objectives, eligibility criteria, sources of evidence, charting methods, results, and conclusions that relate to the review questions and objectives.	1-3
**INTRODUCTION**			
Rationale	3	Describe the rationale for the review in the context of what is already known. Explain why the review questions/objectives lend themselves to a scoping review approach.	3
Objectives	4	Provide an explicit statement of the questions and objectives being addressed with reference to their key elements (e.g., population or participants, concepts, and context) or other relevant key elements used to conceptualize the review questions and/or objectives.	6
**METHODS**			
Protocol and registration	5	Indicate whether a review protocol exists; state if and where it can be accessed (e.g., a Web address); and if available, provide registration information, including the registration number.	6
Eligibility criteria	6	Specify characteristics of the sources of evidence used as eligibility criteria (e.g., years considered, language, and publication status), and provide a rationale.	7
Information sources*	7	Describe all information sources in the search (e.g., databases with dates of coverage and contact with authors to identify additional sources), as well as the date the most recent search was executed.	7
Search	8	Present the full electronic search strategy for at least 1 database, including any limits used, such that it could be repeated.	Additional file 1
Selection of sources of evidence†	9	State the process for selecting sources of evidence (i.e., screening and eligibility) included in the scoping review.	7
Data charting process‡	10	Describe the methods of charting data from the included sources of evidence (e.g., calibrated forms or forms that have been tested by the team before their use, and whether data charting was done independently or in duplicate) and any processes for obtaining and confirming data from investigators.	7-8
Data items	11	List and define all variables for which data were sought and any assumptions and simplifications made.	Not applicable

SECTION	ITEM	PRISMA-ScR CHECKLIST ITEM	REPORTED ON PAGE #
Critical appraisal of individual sources of evidence§	12	If done, provide a rationale for conducting a critical appraisal of included sources of evidence; describe the methods used and how this information was used in any data synthesis (if appropriate).	Not applicable
Synthesis of results	13	Describe the methods of handling and summarizing the data that were charted.	8
**RESULTS**			
Selection of sources of evidence	14	Give numbers of sources of evidence screened, assessed for eligibility, and included in the review, with reasons for exclusions at each stage, ideally using a flow diagram.	9 (Figure 1)
Characteristics of sources of evidence	15	For each source of evidence, present characteristics for which data were charted and provide the citations.	8-16
Critical appraisal within sources of evidence	16	If done, present data on critical appraisal of included sources of evidence (see item 12).	Not applicable
Results of individual sources of evidence	17	For each included source of evidence, present the relevant data that were charted that relate to the review questions and objectives.	8-16
Synthesis of results	18	Summarize and/or present the charting results as they relate to the review questions and objectives.	8-16
**DISCUSSION**			
Summary of evidence	19	Summarize the main results (including an overview of concepts, themes, and types of evidence available), link to the review questions and objectives, and consider the relevance to key groups.	16-19
Limitations	20	Discuss the limitations of the scoping review process.	19
Conclusions	21	Provide a general interpretation of the results with respect to the review questions and objectives, as well as potential implications and/or next steps.	19-20
**FUNDING**			
Funding	22	Describe sources of funding for the included sources of evidence, as well as sources of funding for the scoping review. Describe the role of the funders of the scoping review.	21

Additional File 2: Tailored search strategies for included databases

	MEDLINE (Ovid)	EMBASE (Ovid)	Web of Science	LILACs
1	(cohort$ or longitudinal).ti.	(cohort$ or longitudinal).ti.	TI=(cohort$ or longitudinal).	(ti:((cohort* OR longitudinal))) AND ((ti:((pregnan* OR *pregnan* OR maternal))) OR (((maternal OR mother) near/4 exposure*))) AND (db:("LILACS"))
2	(pregnan$ or $pregnan$ or maternal).ti.	(pregnan$ or $pregnan$ or maternal).ti.	TI=(pregnan$ or $pregnan$ or maternal)	PY=2000–2021
3	((maternal or mother) adj4 exposure*).ti,ab	((maternal or mother) adj4 exposure*).ti,ab	TI=((maternal or mother) NEAR/4 exposure*) OR AB=((maternal or mother) NEAR/4 exposure*)	
4	1 and (2 or 3)	1 and (2 or 3)	1 and (2 OR 3)	
5	exp animals/not humans.sh	exp animals/not humans.sh	TS=(animal model or animal* NOT human)	
6	4 not 5	4 not 5	4 not 5	
7	limit 6 to yr="2000 -Current"	limit 6 to yr="2000 -Current"	PY=(2000–2021)	

Additional File 3: List of excluded studies along with reasons for exclusion

Exclusion reason	Citation	Title	Authors	Published Year	DOI
Did not enroll pregnant women (e.g. enrolled infants at birth)	Imaz 2009	**Substance use during pregnancy and child outcomes: a longitudinal study**	Imaz, M.; Gelabert, E.; Garcia-Esteve, L. L.; Navarro, P.; Sanjuan, J.; Guillamat, R.; Gutierrez, A.; Torrens, M.; Langorh, K.; Martin-Santos, R.	2009	10.1016/s0924-977x(09)71088-0
	Azeredo 2017	**Maternal depression and bullying victimization among adolescents: Results from the 2004 Pelotas cohort study**	Azeredo, Catarina Machado; Santos, Ina S.; Barros, Aluisio J. D.; Barros, Fernando C.; Matijasevich, Alicia	2017	https://dx.doi.org/10.1002/da.22662
	Griffith 2019	**Maternal glycemic control in diabetic pregnancies and neurodevelopmental outcomes in preschool aged children. A prospective cohort study**	Griffith, Rebecca J.; Harding, Jane E.; McKinlay, Christopher J. D.; Wouldes, Trecia A.; Harris, Deborah L.; Alsweiler, Jane M.; Chyld Study Team	2019	https://dx.doi.org/10.1016/j.earlhumdev.2019.01.010
	Hall 2020	**Prenatal maternal infections and children's socioemotional development: findings from the UK Millennium Cohort Study**	Hall, Hildigunnur Anna; Speyer, Lydia Gabriela; Murray, Aja Louise; Auyeung, Bonnie	2020	https://dx.doi.org/10.1007/s00787-020-01644-y
	Jones 2000	**Maternal diet during pregnancy is associated with bone mineral density in children: a longitudinal study**	Jones, G.; Riley, M. D.; Dwyer, T.	2000	
	Stone 2009	**Longitudinal study of maternal report of sleep problems in children with prenatal exposure to cocaine and other drugs**	Stone, Kristen C.; High, Pamela C.; Miller-Loncar, Cynthia L.; Lagasse, Linda L.; Lester, Barry M.	2009	https://dx.doi.org/10.1080/15402000903190108

Exclusion reason	Citation	Title	Authors	Published Year	DOI
	Williams 2020	**Association of maternal antiretroviral use with microcephaly in children who are HIV-exposed but uninfected (SMARTT): a prospective cohort study**	Williams, Paige L.; Yildirim, Cenk; Chadwick, Ellen G.; Van Dyke, Russell B.; Smith, Renee; Correia, Katharine F.; DiPerna, Alexandria; Seage, George R., 3rd; Hazra, Rohan; Crowell, Claudia S.; Surveillance Monitoring for, A. R. T. Toxicities study of the Pediatric H. I. V. Aids Cohort Study	2020	https://dx.doi.org/10.1016/S2352-3018(19)30340-6
	Liu 2017	**Prenatal exposure to maternal bereavement and offspring psoriasis: a Danish nationwide cohort study**	Liu, X.; Chen, J.; Olsen, J.; Schlunssen, V.; Momen, N.; Li, J.	2017	https://dx.doi.org/10.1111/bjd.15224
	Biederman 2017	**Is Maternal Smoking During Pregnancy a Risk Factor for Cigarette Smoking in Offspring? A Longitudinal Controlled Study of ADHD Children Grown Up**	Biederman, Joseph; Martelon, MaryKate; Woodworth, K. Yvonne; Spencer, Thomas J.; Faraone, Stephen V.	2017	https://dx.doi.org/10.1177/1087054714557357
	Maher 2019	**ASSOCIATION BETWEEN HYPERTENSIVE DISORDERS OF PREGNANCY AND ATTENTION DEFICIT HYPERACTIVITY DISORDER: A POPULATION-BASED AND SIBLING-MATCHED COHORT STUDY**	Maher, G. M.; Dalman, C.; Kearney, P. M.; O'Keeffe, G. W.; McCarthy, F. P.; Kenny, L. C.; Khashan, A. S.	2019	10.1136/jech-2019-SSMabstracts.103
Duplicates	Skurtveit 2013	**Prenatal Exposure to Antidepressants and Language Competence at 3 Years of Age. Results from a Large Population Based Pregnancy Cohort in Norway**	Skurtveit, S.; Selmer, R.; Roth, C.; Hernandez-Diaz, S.; Handal, M.	2013	
	Dachew 2020	**Hypertensive disorders of pregnancy and the risk of offspring depression in childhood: Findings from the Avon**	Dachew, Berihun Assefa; Scott, James G.; Betts, Kim; Mamun, Abdullah; Alati, Rosa	2020	https://dx.doi.org/10.1017/S0954579419000944

Exclusion reason	Citation	Title	Authors	Published Year	DOI
		**Longitudinal Study of Parents and Children**			
Not a peer reviewed pub or a systematic review (letter to the editor, commentary, erratum, masters thesis, abstract)	Ozturk 2019	**Exposure to Antidepressants during Pregnancy and the Risk of Attention Deficit Hyperactivity Disorder in Offspring: Findings from a Nationwide Cohort Study with and without Sibling Design**	Ozturk, B.; Pedersen, L.; Ehrenstein, V.; Sorensen, H. T.	2019	
	Zhao 2016	**Impact of hypertensive disorders of pregnancy on adverse outcomes: a 10-year retrospective double cohort study in Shanghai, China**	Zhao, M. M.; Jue, L.	2016	
	Tanaka 2015	**Secondhand smoke and incidence of dental caries in deciduous teeth among children in Japan: population based retrospective cohort study**	Tanaka, S.; Shinzawa, M.; Tokumasu, H.; Seto, K.; Tanaka, S.; Kawakami, K.	2015	10.1136/bmj.h5397
	Montoya-Fernández 2012	**Comentarios sobre el artículo misoprostol intravaginal administrado ambulatoriamente para inducción selectiva del trabajo de parto en pacientes con embarazo a término: estudio de cohorte**	Montoya-Fernández, Jeanette; León-Jiménez, Franco	2012	
	Baird 2017	**PRE-PREGNANCY AND POSTNATAL DEPRESSIVE SYMPTOMS ARE ASSOCIATED WITH QUALITY OF MOTHER-CHILD RELATIONSHIPS; LONGITUDINAL DATA FROM THE SOUTHAMPTON WOMEN'S SURVEY**	Baird, J.; Barker, M.; Lawrence, W.; Kendrick, T.; Crozier, S.; Robinson, S. M.; Cooper, C.; Godfrey, K. M.; Inskip, H. M.	2017	10.1136/jech-2017-SSMAbstracts.88
	Chan 2010	**Neurocognitive development of children following in-utero exposure to labetalol for maternal hypertension: a cohort study using a prospectively collected database**	Chan, Wee Shian; Koren, Gideon; Barrera, Maru; Rezvani, Massoud; Knittel-Keren, Dafna; Nulman, Irena	2010	https://dx.doi.org/10.3109/10641950902777705

Exclusion reason	Citation	Title	Authors	Published Year	DOI
	Fluegge 2016	**Re: Attention deficit hyperactivity disorder and autism spectrum disorder in children born to mothers with thyroid dysfunction: a Danish nationwide cohort study: Maternal hypothyroidism and risk of autism**	Fluegge, Keith	2016	https://dx.doi.org/10.1111/1471-0528.13955
	Furtado 2013	**Alcohol exposure in pregnancy and cognitive development at school age: a Brazilian longitudinal study**	Furtado, E. F.; Alcantara, L. I.	2013	
	Greenbaum 2018	**Maternal smoking during pregnancy and long-term gastrointestinal morbidity in the offspring: Results from a single-center population-based cohort study**	Greenbaum, S.; Wainstock, T.; Walfisch, A.; Landau, D.; Sheiner, E.	2018	
	Grupp-Phelan 2010	**Maternal depression and child growth: definitional issues, longitudinal trajectories, and analytic considerations**	Grupp-Phelan, Jacqueline; Ammerman, Robert T.	2010	https://dx.doi.org/10.1016/j.jpeds.2010.04.061
	Kawada 2016	**Re: Associations of maternal prepregnancy body mass index and gestational weight gain with cardio-metabolic risk factors in adolescent offspring:a prospective cohort study**	Kawada, Tomoyuki	2016	https://dx.doi.org/10.1111/1471-0528.14283
	Kuja-Halkola 2012	**Is smoking during pregnancy dangerous for the offspring? Estimating gene-environment correlation in a children of siblings design using a National Swedish Cohort**	Kuja-Halkola, R.; D'Onofrio, B.; Langstrom, N.; Larsson, H.; Lichtenstein, P.	2012	
	Qamar 2017	**PARATHYROID HORMONE AND FETAL LENGTH IN A PREGNANCY COHORT IN DHAKA, BANGLADESH**	Qamar, H.; Perumal, N.; Papp, E.; Gernand, A. D.; Al Mahmud, A.; Roth, D.	2017	
	Quinlivan 2020	**Will COVID-19 impact upon pregnancy, childhood and adult outcomes? A call to establish national longitudinal datasets**	Quinlivan, Julie; Lambregtse-van den Berg, Mijke	2020	https://dx.doi.org/10.1080/0167482X.2020.1775925

Exclusion reason	Citation	Title	Authors	Published Year	DOI
	Rae 2019	**The Association between Maternal Adiposity and Offspring Early Childhood Kidney Function in an Indigenous Australian Pregnancy-through-to-Early-Childhood Cohort Study: The Gomeroi Gaaynggal Study**	Rae, K. M.; Schumacher, T.; Lumbers, E. R.; Lee, Y. Q.; Keogh, L.; Sutherland, K.; Weatherall, L.; Pringle, K. G.	2019	
	Reyes 2010	**Maternal Demoralization Is Associated with Childhood Wheeze, But Not IgE, at Age 5 Years in an Inner-City Cohort**	Reyes, M.; Perzanowski, M. S.; Whyatt, R. M.; Kelvin, E.; Diaz, D. M.; Plaza, M. B.; Hoepner, L. A.; Perera, F. P.; Rauh, V. A.; Miller, R. L.	2010	10.1016/j.jaci.2009.12.819
	Watson 2003	**Maternal influenza and schizophrenia: A cohort study**	Watson, J. B.; Mednick, S. A.; Machon, R.	2003	10.1016/s0920-9964(03)80549-2
	Webster 2017	**Re: Chronic hypertension and adverse pregnancy outcome: a cohort study. A. M. Panaitescu, A. Syngelaki, N. Prodan, R. Akolekar and K. H. Nicolaides. Ultrasound Obstet Gynecol 2017; 50: 228-235**	Webster, L. M.	2017	https://dx.doi.org/10.1002/uog.17554
	Zhang 2021	**Correction to: Maternal HBsAg carriers and pregnancy outcomes: a retrospective cohort analysis of 85,190 pregnancies**	Zhang, Yulong; Chen, Jiacheng; Liao, Tingting; Chen, Siwen; Yan, Jianying; Lin, Xiaoqian	2021	https://dx.doi.org/10.1186/s12884-021-03630-x
	Ask 2019	**EXPLORING THE ASSOCIATIONS BETWEEN HEALTH-RELATED POLYGENIC SCORES AND PARTICIPATION IN THE NORWEGIAN MOTHER AND CHILD PREGNANCY COHORT (MOBA)**	Ask, H.; Hannigan, L.; Davies, N.; Gustavson, K.; Biele, G.; Richardson, T.; Cornish, R.; Corfield, E.; Askeland, R. B.; Tesli, M.; Tilling, K.; Smith, G. D.; Magnus, P.; Reichborn-Kjennerud, T.; Havdahl, A.	2019	10.1016/j.euroneuro.2019.08.170

Exclusion reason	Citation	Title	Authors	Published Year	DOI
	Wigal 2011	**Longitudinal Growth Curves and Adolescent Height in Children with ADHD: Effects of Maternal Smoking during Pregnancy and a Decade of Treatment with Stimulant Medication**	Wigal, T.; Stehli, A.; Swanson, J.	2011	
	Agbota 2019	**MATERNAL URINARY SCHISTOSOMIASIS AND MALARIA BEFORE CONCEPTION AND DURING PREGNANCY ARE ASSOCIATED WITH CHILD'S EARLY HAEMOGLOBIN CONCENTRATIONS AND RISK OF INFECTIONS: A LONGITUDINAL PRECONCEPTIONAL COHORT IN BENIN**	Agbota, G.; Polman, K.; Wieringa, F.; Campos-Ponce, M.; Fievet, N.; Accrombessi, M.; Yovo, E.; Roucher, C.; Pachot, A.; Ganee, L.; Tissieres, P.; Massougbodji, A.; Cot, M.; Briand, V.	2019	
	Levey 2020	**MATERNAL TRAUMA AND OFFSPRING BEHAVIORAL PROBLEMS: FINDINGS FROM A COHORT STUDY IN PERU**	Levey, E. J.	2020	10.1016/j.jaac.2020.07.635
	Mattes 2007	**The influence of mental health in early childhood on cardiovascular risk factors at age 8 in the Western Australian Pregnancy Cohort (Raine) Study**	Mattes, E.; Huang, R. C.; Jacoby, R.; Kendall, G. E.; Susser, E. S.; McKeague, I. W.; Zubrick, S. R.; Silburn, S. R.; Stanley, F. J.; Beilin, L. J.	2007	10.1016/s0378-3782(07)70203-7
	Nezvalova-Henriksen 2014	**Acetaminophen Exposure during Pregnancy and Childhood Asthmatic Symptoms: Results from the Norwegian Mother and Child Cohort Study (MoBa) Cohort Study**	Nezvalova-Henriksen, K.; Brandlistuen, R. E.; Spigset, O.; Koren, G.; Nordeng, H. M. E.	2014	
	Clausen 2017	**Pregnancy and cord vitamin D status and symptoms of autism spectrum disorders in toddlers: An Odense child cohort study**	Clausen, S. S. B.; Christesen, H. T.; Dalgard, C.; Lykkedegn, S.; Munk-Jorgensen, P.; Bilenberg, N.	2017	10.1016/j.eurpsy.2017.01.1937

Exclusion reason	Citation	Title	Authors	Published Year	DOI
	Santorelli 2018	**Ethnic differences in the association between maternal vitamin D status and offspring asthma and wheeze: Findings from the Born in Bradford cohort study**	Santorelli, G.; Wright, J.; Sheikh, A.	2018	https://dx.doi.org/10.1111/all.13447
	Dobano 2011	**Immune responses to Plasmodium falciparum in a cohort of Mozambican pregnant women and their children in relation to age, exposure and clinical outcomes**	Dobano, C.; Mayor, A. G.; Nhabomba, A.; Manaca, N.; Guinovart, C.; Berthoud, T.; Quinto, L.; Aguilar, R.; Barbosa, A.; Jimenez, A.; Bassat, Q.; Aponte, J. J.; Chitnis, C.; Doolan, D. L.; Alonso, P. L.	2011	
	Boukhris 2014	**ANTIDEPRESSANT USE DURING PREGNANCY AND RISK OF AUTISM SPECTRUM DISORDERS IN CHILDREN: A POPULATION-BASED COHORT STUDY**	Boukhris, T.; Sheehy, O.; Berard, A.	2014	10.1016/j.jval.2014.03.1236
	Magnus 2013	**Maternal Vitamin D Status During Pregnancy and Asthma in the Offspring Among Participants in the Norwegian Mother and Child Cohort Study**	Magnus, M. C.; Nafstad, P.; Stene, L. C.; Haberg, S. E.; London, S. J.; Stigum, H.; Nystad, W.	2013	10.1016/j.jaci.2012.12.1126
	Navarro 2020	**Associations between maternal healthy lifestyle score and offspring birth outcomes and childhood obesity: results from the Lifeways Cross-Generation Cohort Study**	Navarro, P.; Mehegan, J.; Murrin, C.; Kelleher, C.; Phillips, C.	2020	10.1017/s0029665120005467
	Pesce 2015	**Fetal exposure to pregnancy complications and respiratory health in childhood: A longitudinal study**	Pesce, G.; Marcon, A.; Marchetti, P.; Calciano, L.; Guarda, L.; Ricci, P.; Pironi, V.; De Marco, R.	2015	10.1183/13993003.congress-2015.OA4765
	Totsika 2012	**Longitudinal relationship between maternal well-being and child behaviour in young children with ASD**	Totsika, V.; Hastings, R. P.; Emerson, E.; Berridge, D. M.; Lancaster, G. A.	2012	

Exclusion reason	Citation	Title	Authors	Published Year	DOI
	Chuang 2009	**Herbal medicines during pregnancy and childhood cancers: an analysis of data from a pregnancy cohort study**	Chuang, Chao-Hua; Doyle, Pat; Wang, Jung-Der; Chang, Pei-Jen; Lai, Jung-Nien; Chen, Pau-Chung	2009	https://dx.doi.org/10.1002/pds.1835
	Lu 2018	**Examine the relationship between maternal vitamin D supplementation during pregnancy and eczema in their offspring in a cohort study of children aged 6 years**	Lu, K.; Huang, Y.; Huang, Y.; Tsai, Z.; Chiu, C.; Lin, C.; Fang, H.; Kao, P.; Lee, W.; Gau, C.; Tsai, H.; Yao, T.	2018	
	Tan 2019	**Association between Maternal Overweight and Obesity with Offspring Neurodevelopment: Findings from Pregnancy Cohort Study Universiti Sains Malaysia**	Tan, S. Y.; Mohamed, N. N.; Loy, S. L.; Lim, P. Y.; Mohamed, H. J. J.	2019	
	Li 2020	**Impact of maternal depression during pregnancy and its treatment options on fetal health: A prospective cohort study of 84,000 pregnant women**	Li, D. K.; Ferber, J. R.; Odouli, R.; Quesenberry, C.; Avalos, L. A.; Turner, M.; Flanagan, T.	2020	
	Mattera 2019	**LONGITUDINAL AND MULTIMODAL SLEEP ASSESSMENT IN CHILDREN OF MOTHERS WITH DEPRESSION DURING PREGNANCY: PRENATAL ANTIDEPRESSANT EXPOSURE ASSOCIATED WITH DECREASED SLEEP DURATION AT 18-MONTHS AND EARLIER SLEEP SCHEDULES AT 18-AND 36-MONTHS**	Mattera, J. A.; Stone, K. C.; Salisbury, A. L.	2019	
	Santana 2013	**Consumo alimentar na gestação e ganho ponderal: um estudo de coorte de gestantes da zona oeste do município de São Paulo**	Santana, Andreia Cardoso de	2013	

Exclusion reason	Citation	Title	Authors	Published Year	DOI
	Maugeri 2017	**Mediterranean diet, nutrient intakes and pregnancy: results from a Sicilian mother-child cohort**	Maugeri, A. G.; Barchitta, M.; Quattrocchi, A.; La Rosa, M. C.; Caruso, M.; Panella, M.; Cianci, A.; Agodi, A.	2017	
	Chatzi 2013	**Maternal glucose levels in early pregnancy, gestational diabetes, prepregnancy BMI and the risk of childhood obesity: the mother child cohort, RHEA study in Crete, Greece**	Chatzi, L.; Karachaliou, M.; Daraki, V.; Georgiou, V.; Koinaki, S.; Chalkiadaki, G.; Pappas, A.; Kogevinas, M.	2013	
	White 2014	**Early life risk factors for asthma in young adults in the Western Australian pregnancy cohort Maine study)**	White, E.; DeKlerk, N.; Hollams, E.; Holt, P.; Sly, P.; Hall, G.	2014	
	Hardy 2006	**Maternal valproate in pregnancy and risk of childhood language disorders in a cohort of 81,975 mother-baby pairs**	Hardy, J. R.; Adams, J.; Crawford, S. L.; Kelsey, J. L.	2006	
	Brion 2011	**Maternal Prenatal Smoking and Child Aggression: Exploring Intrauterine Effects in UK, Australian and Brazilian Cohorts**	Brion, M. J. A.; Robinson, M.; Matijasevich, A.; Steer, C.; Anselimi, L.; Menezes, A. M. B.; Pennell, C.; Palmer, L. J.; Victora, C. G.; Smith, G. D.; Lawlor, D. A.	2011	
	Hemmingsen 2020	**Maternal Use of Hormonal Contraception and Risk of Childhood Cancer: A Nationwide Population-Based Cohort Study**	Hemmingsen, C.; Hargreave, M.; Morch, L.; Kjaer, S.; Winther, J.	2020	
	Shrivastava 2010	**Relationship between maternal BMI, maternal diet and child BMI at 6-year follow-up in lifeways cross generation cohort study**	Shrivastava, A.; Murrin, C.; O'Mahony, D.; Kelleher, C. C.; Lifeways Cross Generation Cohort, S.	2010	

Exclusion reason	Citation	Title	Authors	Published Year	DOI
	Taylor-Robinson 2014	**HOW DOES MATERNAL SMOKING DURING PREGNANCY AND BREASTFEEDING INFLUENCE INEQUALITIES IN WHEEZING IN CHILDREN? FINDINGS FROM THE UK MILLENNIUM COHORT STUDY**	Taylor-Robinson, D.; Smyth, R.; Law, C.; Pearce, A.	2014	10.1136/jech-2014-204726.92
	Rudge 2019	**PRE-PREGNANCY METABOLIC SYNDROME ON SHORT AND LONG-TERM ADVERSE CONSEQUENCES FOR THE OFFSPRING: A COHORT STUDY OF LOW INCOME BRAZILIAN MOTHERS**	Rudge, M. V. C.; de Camargo, L. P.; Arantes, M. A.; Negrato, C. A.; Bossolan, G.; Hallur, L. S. R.; Magalhaes, C. G.; Costa, R.; Abbade, J. F.; Ferraz, G. A. R.; Damasceno, D. C.; Calderon, I. D. P.; Barbosa, A. M. P.	2019	
	Tam 2018	**The association of maternal gestational weight gain with children's insulin resistance and beta cell function - Follow-up of the HAPO cohort**	Tam, W. H.; Tam, H. T.; Ma, R. C. W.; Yuen, L. Y.; Ng, A. C. W.; Chan, J. C. N.	2018	
	Ziauddeen 2019	**Maternal interpregnancy weight change and childhood overweight and obesity: findings from a UK population-based cohort**	Ziauddeen, N.; Alwan, N. A.	2019	
	Lawlor 2011	**HYPERTENSIVE DISORDERS OF PREGNANCY AND OFFSPRING VASCULAR, INFLAMMATORY AND LIPID OUTCOMES IN CHILDHOOD: FINDINGS FROM THE AVON LONGITUDINAL STUDY OF PARENTS AND CHILDREN**	Lawlor, D. A.; Macdonald-Wallis, C.; Fraser, A.; Nelson, S. M.; Hingorani, A.; Smith, G. D.; Sattar, N.; Deanfield, J.	2011	10.1136/jech.2011.142976a.22
Pregnancy cohort up to 2 years	Montastruc 2015	**Atropinic Burden of Medications During Pregnancy and Psychological Development of the Child: A Cohort Study in EFEMERIS**	Montastruc, J. L.; Beau, A. B.; Lacroix, I.; Montastruc, F.; Hurault-Delarue, C.; Damase-Michel, C.	2015	

Exclusion reason	Citation	Title	Authors	Published Year	DOI
	Madsen 2017	**Pregnancy exposure to air pollution and early childhood respiratory health in the Norwegian Mother and Child Cohort Study (MoBa)**	Madsen, Christian; Haberg, Siri Eldevik; Magnus, Maria C.; Aamodt, Geir; Stigum, Hein; London, Stephanie J.; Nystad, Wenche; Nafstad, Per	2017	https://dx.doi.org/10.1136/bmjopen-2016-015796
	Babu 2014	**Effect of hyperglycaemia in pregnancy on adiposity in their infants in India: a protocol of a multicentre cohort study**	Babu, Giridhara R.; Garadi, Lavanya; Murthy, G. V. S.; Kinra, Sanjay	2014	https://dx.doi.org/10.1136/bmjopen-2014-005417
	Chatzi 2012	**Effect of high doses of folic acid supplementation in early pregnancy on child neurodevelopment at 18 months of age: the mother-child cohort 'Rhea' study in Crete, Greece**	Chatzi, Leda; Papadopoulou, Eleni; Koutra, Katerina; Roumeliotaki, Theano; Georgiou, Vaggelis; Stratakis, Nikolaos; Lebentakou, Vassiliki; Karachaliou, Mariana; Vassilaki, Maria; Kogevinas, Manolis	2012	https://dx.doi.org/10.1017/S1368980012000067
	Gonçalves 2003	**Avaliação longitudinal da infecção por parvovírus B19 entre grávidas em Ribeirão Preto, SP, Brasil**	Gonçalves, Carla Vitola; Duarte, Geraldo; Marcolin, Alessandra Cristina; Quintana, Silvana Maria; Covas, Dimas Tadeu; Costa, Juvenal Soares Dias da	2003	

Exclusion reason	Citation	Title	Authors	Published Year	DOI
	Hutchinson 2018	**Cohort Profile: The Triple B Pregnancy Cohort Study: A longitudinal study of the relationship between alcohol, tobacco and other substance use during pregnancy and the health and well-being of Australian children and families**	Hutchinson, Delyse; Wilson, Judy; Allsop, Steve; Elliott, Elizabeth; Najman, Jake; Burns, Lucinda; Bartu, Anne; Jacobs, Sue; Honan, Ingrid; McCormack, Clare; Rossen, Larissa; Fiedler, Hannah; Stone, Chiara; Khor, Sarah; Ryan, Joanne; J Youssef, George; A Olsson, Craig; P Mattick, Richard; Triple, B. Research Consortium	2018	https://dx.doi.org/10.1093/ije/dyx126
	Lee 2018	**Effects of Maternal Iodine Status during Pregnancy and Lactation on Maternal Thyroid Function and Offspring Growth and Development: A Prospective Study Protocol for the Ideal Breast Milk Cohort**	Lee, Young Ah; Cho, Sun Wook; Sung, Ho Kyung; Kim, Kyungsik; Song, Young Shin; Moon, Sin Je; Oh, Jung Won; Ju, Dal Lae; Choi, Sooyeon; Song, Sang Hoon; Cheon, Gi Jeong; Park, Young Joo; Shin, Choong Ho; Park, Sue K.; Jun, Jong Kwan; Chung, June Key	2018	https://dx.doi.org/10.3803/EnM.2018.33.3.395
	Leslie 2020	**Is Anemia in Low Income Pregnant Women Related to Their Infants' Having Anemia? A Cohort Study of Pregnant Women-Infant Pairs in the United States**	Leslie, Mayri Sagady; Park, Jeongyoung; Briggs, Linda A.; El-Banna, Majeda M.; Greene, Jessica	2020	https://dx.doi.org/10.1007/s10995-020-02912-8

Exclusion reason	Citation	Title	Authors	Published Year	DOI
	Lewi 2008	**PREGNANCY AND LONG-TERM NEURODEVELOPMENTAL OUTCOME IN MONOCHORIONIC DIAMNIOTIC TWIN PREGNANCIES: A MULTI-CENTER PROSPECTIVE COHORT STUDY**	Lewi, L.; Ortibus, E.; Lopriore, E.; Done, E.; Jani, J.; Van Mieghem, T.; Diemert, A.; Gucciardo, L.; Klaritsch, P.; Hecher, K.; De Cock, P.; Lagae, L.; Deprest, J.	2008	10.1016/j.ajog.2008.09.102
	Nath 2017	**Effect of prenatal exposure to maternal cortisol and psychological distress on infant development in Bengaluru, southern India: a prospective cohort study**	Nath, Anita; Murthy, Gudlavalleti Venkata Satyanarayana; Babu, Giridhara R.; Di Renzo, Gian Carlo	2017	https://dx.doi.org/10.1186/s12888-017-1424-x
	Phua 2017	**Positive maternal mental health during pregnancy associated with specific forms of adaptive development in early childhood: Evidence from a longitudinal study**	Phua, Desiree Y.; Kee, Michelle K. Z. L.; Koh, Dawn X. P.; Rifkin-Graboi, Anne; Daniels, Mary; Chen, Helen; Chong, Yap Seng; Broekman, Birit F. P.; Magiati, Iliana; Karnani, Neerja; Pluess, Michael; Meaney, Michael J.; Growing Up In Singapore Towards Healthy Outcomes Study, Group	2017	https://dx.doi.org/10.1017/S0954579417001249
	Polanska 2016	**Selenium status during pregnancy and child psychomotor development-Polish Mother and Child Cohort study**	Polanska, Kinga; Krol, Anna; Sobala, Wojciech; Gromadzinska, Jolanta; Brodzka, Renata; Calamandrei, Gemma; Chiarotti, Flavia; Wasowicz, Wojciech; Hanke, Wojciech	2016	https://dx.doi.org/10.1038/pr.2016.32

Exclusion reason	Citation	Title	Authors	Published Year	DOI
	Polanska 2015	**Maternal lifestyle during pregnancy and child psychomotor development - Polish Mother and Child Cohort study**	Polanska, Kinga; Muszynski, Pawel; Sobala, Wojciech; Dziewirska, Emila; Merecz-Kot, Dorota; Hanke, Wojciech	2015	https://dx.doi.org/10.1016/j.earlhumdev.2015.03.002
	Soto-Ramirez 2012	**Maternal immune markers in serum during gestation and in breast milk and the risk of asthma-like symptoms at ages 6 and 12 months: a longitudinal study**	Soto-Ramirez, Nelis; Karmaus, Wilfried; Yousefi, Mitra; Zhang, Hongmei; Liu, Jihong; Gangur, Venugopal	2012	https://dx.doi.org/10.1186/1710-1492-8-11
	Sutter-Dallay 2015	**The MATQUID Cohort: Links Between Maternal Depressive Symptoms and Children's Developmental Trajectories**	Sutter-Dallay, A.	2015	10.1016/s0924-9338(15)30087-0
	Zhang 2020	**Associations of maternal glycemia and prepregnancy BMI with early childhood growth: a prospective cohort study**	Zhang, Yaqi; Chen, Zhong; Cao, Zhongqiang; Zhang, Yiming; Yao, Cong; Qiu, Lin; Li, Yuanyuan; Xu, Shunqing; Zhou, Aifen; Xia, Wei	2020	https://dx.doi.org/10.1111/nyas.14258
	Millard 2013	**Physical activity during pregnancy and offspring cardiovascular risk factors: findings from a prospective cohort study (vol 3, e003574, 2013)**	Millard, L. A. C.; Lawlor, D. A.; Fraser, A.	2013	10.1136/bmjopen-2013-003574corr1
	Henriksen 2015	**Marital Quality and Stress in Pregnancy Predict the Risk of Infectious Disease in the Offspring: The Norwegian Mother and Child Cohort Study**	Henriksen, Roger Ekeberg; Thuen, Frode	2015	https://dx.doi.org/10.1371/journal.pone.0137304

Exclusion reason	Citation	Title	Authors	Published Year	DOI
Pregnancy cohort with no child outcomes or other wrong outcome	Zhang 2020	**Effect of previous placenta previa on outcome of next pregnancy: a 10-year retrospective cohort study**	Zhang, Lizi; Bi, Shilei; Du, Lili; Gong, Jingjin; Chen, Jingsi; Sun, Wen; Shen, Xinyang; Tang, Jingman; Ren, Luwen; Chai, Guolu; Wang, Zhijian; Chen, Dunjin	2020	https://dx.doi.org/10.1186/s12884-020-02890-3
	Woolner 2018	**Second pregnancy outcomes following initial third or fourth degree tear: A retrospective population-based cohort study of 182 445 women in Scotland**	Woolner, A.; Ayansina, D.; Bhattacharya, S.; Black, M.	2018	
	Smith 2015	**The Association Between Maternal Depression During Pregnancy and Adverse Birth Outcomes: A Retrospective Cohort Study of PRAMS Participants**	Smith, Kenesha F.; Huber, Larissa R. Brunner; Issel, L. Michele; Warren-Findlow, Jan	2015	https://dx.doi.org/10.1007/s10900-015-0022-4
	Smith 2007	**Previous preeclampsia, preterm delivery, and delivery of a small for gestational age infant and the risk of unexplained stillbirth in the second pregnancy: a retrospective cohort study, Scotland, 1992-2001**	Smith, Gordon C. S.; Shah, Imran; White, Ian R.; Pell, Jill P.; Dobbie, Richard	2007	
	Smith 2007	**Maternal obesity in early pregnancy and risk of spontaneous and elective preterm deliveries: a retrospective cohort study**	Smith, Gordon C. S.; Shah, Imran; Pell, Jill P.; Crossley, Jennifer A.; Dobbie, Richard	2007	
	Nascimento 2017	**Symptomatic dengue infection during pregnancy and livebirth outcomes in Brazil, 2007-13: a retrospective observational cohort study**	Nascimento, Laura B.; Siqueira, Claudio M.; Coelho, Giovanini E.; Siqueira, Joao B., Jr.	2017	https://dx.doi.org/10.1016/S1473-3099(17)30169-X
	Mahande 2016	**Effect of interpregnancy interval on adverse pregnancy outcomes in northern Tanzania: a registry-based retrospective cohort study**	Mahande, Michael J.; Obure, Joseph	2016	https://dx.doi.org/10.1186/s12884-016-0929-5

Exclusion reason	Citation	Title	Authors	Published Year	DOI
	Long 2020	**Pregnancy Outcomes of Single/Double Blastocysts and Cleavage Embryo Transfers: a Retrospective Cohort Study of 24,422 Frozen-Thawed Cycles**	Long, Xiaoyu; Wang, Yuanyuan; Wu, Fangrong; Li, Rong; Chen, Lixue; Qian, Weiping; Qiao, Jie	2020	https://dx.doi.org/10.1007/s43032-020-00247-x
	McCarthy 2015	**Long-term safety of alemtuzumab in relapsing-remitting multiple sclerosis: pregnancy and infection data from a cohort of patients on open label studies in Cambridge, UK**	McCarthy, C. L.; Brown, J. W. L.; Tuohy, O.; Azzopardi, L.; Kousin-Ezewu, O.; Jones, J. L.; Compston, A.; Coles, A. J.	2015	
	McBain 2016	**Impact of inter-pregnancy BMI change on perinatal outcomes: a retrospective cohort study**	McBain, Rosemary D.; Dekker, Gustaaf A.; Clifton, Vicki L.; Mol, Ben W.; Grzeskowiak, Luke E.	2016	https://dx.doi.org/10.1016/j.ejogrb.2016.07.487
	Poda 2016	**Pregnancy rate and birth outcomes among women receiving antiretroviral therapy in Burkina Faso: a retrospective cohort study**	Poda, Armel; Hema, Arsene; Konate, Aina; Kabore, Firmin; Zoungrana, Jacques; Kamboule, Euloges; Sore, Ibrahim; Bado, Guillaume; Ouedraogo, Abdoul-Salam; Ouedraogo, Macaire; Meda, Nicolas; Sawadogo, Adrien Bruno	2016	https://dx.doi.org/10.11604/pamj.2016.23.105.7372
	Allswede 2020	**Cytokine concentrations throughout pregnancy and risk for psychosis in adult offspring: a longitudinal case-control study**	Allswede, Dana M.; Yolken, Robert H.; Buka, Stephen L.; Cannon, Tyrone D.	2020	https://dx.doi.org/10.1016/S2215-0366(20)30006-7
	Asselmann 2016	**Peripartum changes in partnership quality among women with and without anxiety and depressive disorders prior to**	Asselmann, E.; Wittchen, H. U.; Petzoldt, J.; Martini, J.	2016	https://dx.doi.org/10.1007/s00737-015-0556-6

Exclusion reason	Citation	Title	Authors	Published Year	DOI
		**pregnancy: a prospective-longitudinal study**			
	Barnett 2018	**Maltreatment in childhood and intimate partner violence: A latent class growth analysis in a South African pregnancy cohort**	Barnett, Whitney; Halligan, Sarah; Heron, Jon; Fraser, Abigail; Koen, Nastassja; Zar, Heather J.; Donald, Kirsty A.; Stein, Dan J.	2018	https://dx.doi.org/10.1016/j.chiabu.2018.08.020
	Blomqvist 2019	**Platelet aggregation in healthy women during normal pregnancy - a longitudinal study**	Blomqvist, Lennart Rune Fredrik; Strandell, Annika Margareta; Baghaei, Fariba; Hellgren, Margareta Sofia Elisabet	2019	https://dx.doi.org/10.1080/09537104.2018.1492106
	BuckLouis 2016	**Lifestyle and pregnancy loss in a contemporary cohort of women recruited before conception: The LIFE Study**	Buck Louis, Germaine M.; Sapra, Katherine J.; Schisterman, Enrique F.; Lynch, Courtney D.; Maisog, Jose M.; Grantz, Katherine L.; Sundaram, Rajeshwari	2016	https://dx.doi.org/10.1016/j.fertnstert.2016.03.009
	DelosRíos 2007	**Fertilidad después del tratamiento laparoscópico del embarazo ectópico, en la Clínica del Prado en Medellín, Colombia. Estudio de cohorte**	De los Ríos, José F.; Sánchez, Enrique; Matta, Gabriel J.; Jaramillo, Mauricio; Arango, Adriana M.	2007	
	Jamnadass 2015	**The perinatal androgen to estrogen ratio and autistic-like traits in the general population: a longitudinal pregnancy cohort study**	Jamnadass, Esha S. L.; Keelan, Jeffrey A.; Hollier, Lauren P.; Hickey, Martha; Maybery, Murray T.; Whitehouse, Andrew J. O.	2015	https://dx.doi.org/10.1186/s11689-015-9114-9

Exclusion reason	Citation	Title	Authors	Published Year	DOI
	Korevaar 2016	**Risk factors and a clinical prediction model for low maternal thyroid function during early pregnancy: two population-based prospective cohort studies**	Korevaar, Tim I. M.; Nieboer, Daan; Bisschop, Peter H. L. T.; Goddijn, Mariette; Medici, Marco; Chaker, Layal; de Rijke, Yolanda B.; Jaddoe, Vincent W. V.; Visser, Theo J.; Steyerberg, Ewout W.; Tiemeier, Henning; Vrijkotte, Tanja G.; Peeters, Robin P.	2016	https://dx.doi.org/10.1111/cen.13153
	Lantsberg 2015	**Is there a disparity in the rate of pregnancy complications among patients diagnosed with cancers prevalent during child-bearing age? Evidence from a large population-based cohort study**	Lantsberg, D.; Yinon, Y.; Dulitzky, M.; Sivan, E.; Shif, E.; Mazaki-Tovi, S.	2015	10.1016/j.ajog.2014.10.504
	Morton 2013	**Pregnancy outcomes of women with physical disabilities: a matched cohort study**	Morton, Christina; Le, Joan T.; Shahbandar, Lena; Hammond, Cassing; Murphy, Eileen A.; Kirschner, Kristi L.	2013	https://dx.doi.org/10.1016/j.pmrj.2012.10.011
	Murphy 2014	**A prospective cohort study of alcohol exposure in early and late pregnancy within an urban population in Ireland**	Murphy, Deirdre J.; Dunney, Clare; Mullally, Aoife; Adnan, Nita; Fahey, Tom; Barry, Joe	2014	https://dx.doi.org/10.3390/ijerph110202049

Exclusion reason	Citation	Title	Authors	Published Year	DOI
	Murray-Davis 2019	**Weight gain during pregnancy: Does the antenatal care provider make a difference? A retrospective cohort study**	Murray-Davis, Beth; Berger, Howard; Melamed, Nir; Hasan, Haroon; Mawjee, Karizma; Syed, Maisah; Ray, Joel G.; Geary, Michael; Barrett, Jon; McDonald, Sarah D.; Diabetes, Obesity; Hypertension in Pregnancy Research, Network; Southern Ontario Obstetrical Network, investigators	2019	https://dx.doi.org/10.9778/cmajo.20180116
	Nakayama 2020	**Hypertensive disorders of pregnancy are associated with dysmenorrhea in early adulthood: A cohort study**	Nakayama, Midori; Ono, Masanori; Iizuka, Takashi; Kagami, Kyosuke; Fujiwara, Tomoko; Sekizuka-Kagami, Naomi; Maida, Yoshiko; Obata, Takeshi; Yamazaki, Rena; Daikoku, Takiko; Fujiwara, Hiroshi	2020	https://dx.doi.org/10.1111/jog.14431
	Padhan 2016	**Temporal relationship of pregnancy to long-term disease course and pregnancy and foetal outcomes: North Indian inflammatory bowel disease cohort study**	Padhan, R. K.; Kumar, S.; Kedia, S.; Dhingra, R.; Mouli, V. P.; Bopanna, S.; Shrivastav, S.; Kurrey, L.; Sreenivas, V.; Makharia, G.; Ahuja, V.	2016	

Exclusion reason	Citation	Title	Authors	Published Year	DOI
	Ramsay 2016	**Smoking in pregnancy, adolescent mental health and cognitive performance in young adult offspring: results from a matched sample within a Finnish cohort**	Ramsay, Hugh; Barnett, Jennifer H.; Murray, Graham K.; Maki, Pirjo; Hurtig, Tuula; Nordstrom, Tanja; Miettunen, Jouko; Kiviniemi, Vesa; Niemela, Solja; Pausova, Zdenka; Paus, Tomas; Veijola, Juha	2016	
	Regan 2015	**A prospective cohort study comparing the reactogenicity of trivalent influenza vaccine in pregnant and non-pregnant women**	Regan, Annette K.; Tracey, Lauren; Blyth, Christopher C.; Mak, Donna B.; Richmond, Peter C.; Shellam, Geoffrey; Talbot, Caroline; Effler, Paul V.	2015	https://dx.doi.org/10.1186/s12884-015-0495-2
	Rodriguez 2019	**Marijuana use in young mothers and adverse pregnancy outcomes: a retrospective cohort study**	Rodriguez, C. E.; Sheeder, J.; Allshouse, A. A.; Scott, S.; Wymore, E.; Hopfer, C.; Hermesch, A.; Metz, T. D.	2019	https://dx.doi.org/10.1111/1471-0528.15885
	Rotem 2020	**Maternal and Neonatal Outcomes Following Trial of Labor After Two Previous Cesareans: a Retrospective Cohort Study**	Rotem, Reut; Hirsch, Ayala; Sela, Hen Y.; Samueloff, Arnon; Grisaru-Granovsky, Sorina; Rottenstreich, Misgav	2020	https://dx.doi.org/10.1007/s43032-020-00378-1
	Rottenstreich 2019	**Risk factors, clinical course and outcomes of pregnancy-related group A streptococcal infections: retrospective 13-year cohort study**	Rottenstreich, A.; Benenson, S.; Levin, G.; Kleinstern, G.; Moses, A. E.; Amit, S.	2019	https://dx.doi.org/10.1016/j.cmi.2018.10.002

Exclusion reason	Citation	Title	Authors	Published Year	DOI
	Santi 2019	**The PLART study: incidence of preterm labor and adverse pregnancy outcomes after assisted reproductive techniques-a retrospective cohort study**	Santi, Erica; Nencini, Giulia; Cerni, Angelica; Greco, Pantaleo; Spelzini, Federico; Tormettino, Beniamino; Scioscia, Marco	2019	https://dx.doi.org/10.1007/s00404-019-05261-2
	Shub 2019	**Timing of diagnosis of gestational diabetes and pregnancy outcomes: A retrospective cohort**	Shub, Alexis; Chee, Tess; Templeton, Alexandra; Boyce, Deborah; McNamara, Catharine; Houlihan, Christine; Churilov, Leonid; McCarthy, Elizabeth A.	2019	https://dx.doi.org/10.1111/ajo.12814
	Sirichotiyakul 2016	**Pregnancy Outcomes Among Women with Homozygous Hemoglobin E Disease: A Retrospective Cohort Study**	Sirichotiyakul, Supatra; Jatavan, Phudit; Traisrisilp, Kuntharee; Tongsong, Theera	2016	
	Smith 2001	**Teenage pregnancy and risk of adverse perinatal outcomes associated with first and second births: population based retrospective cohort study**	Smith, G. C.; Pell, J. P.	2001	
	Tan 2020	**Development and validation of a prediction model on severe maternal outcomes among pregnant women with pre-eclampsia: a 10-year cohort study**	Tan, Jing; Yang, Min; Liao, Yuan; Qi, Yana; Ren, Yan; Liu, Chunrong; Huang, Shiyao; Thabane, Lehana; Liu, Xinghui; Sun, Xin	2020	https://dx.doi.org/10.1038/s41598-020-72527-0

Exclusion reason	Citation	Title	Authors	Published Year	DOI
	Tavares 2009	**Associação entre o padrão de atividade física materna, ganho ponderal gestacional e peso ao nascer em uma coorte de 118 gestantes no município de Campina Grande, Nordeste do Brasil**	Tavares, Jousilene de Sales; Melo, Adriana Suely de Oliveira; Amorim, Melania Maria Ramos de; Benício, Maria Helena D.; Aquino,; Takito, Monica Yuri; Cardoso, Maria Aparecida Alves	2009	
	Thiengo 2012	**Depressão durante a gestação e os desfechos na saúde do recém-nascido: coorte de mães atendidas em unidade básica de saúde**	Thiengo, Daianna Lima; Pereira, Priscila Krauss; Santos, Jacqueline Fernandes de Cintra; Cavalcanti, Maria Tavares; Lovisi, Giovanni Marcos	2012	
	VasalloPrieto 2017	**Comparación de embarazos ectópicos tubarios y cirugía laparoscópica por incisión transversal con la incisión longitudinal**	Vasallo Prieto, Raúl; Sarduy Nápoles, Miguel R.; Díaz Rondón, Belsis; Reyes Llerena, Gil Alberto; del Valle Alonso, Orlando; Molina Peñate, Lisbet	2017	
	Villanueva 2006	**[Measurement of drinking water contaminants and water use activities during pregnancy in a cohort study in Spain]**	Villanueva, Cristina M.; Grimalt, Joan O.; Ballester, Ferran; Ibarluzea, Jesus; Sala, Maria; Tardon, Adonina; Romero-Aliaga, Elena; Fernandez, Marieta; Ribas-Fito, Nuria; Kogevinas, Manolis	2006	

Exclusion reason	Citation	Title	Authors	Published Year	DOI
	Yoshizato 2010	**Longitudinal changes in canal length at 16-35 weeks in normal twin pregnancies and twin pregnancies with preterm labor and delivery**	Yoshizato, Toshiyuki; Inoue, Yoshihito; Fukami, Tatsuya; Sanui, Ayako; Miyamato, Shingo; Kawarabayashi, Tatsuhiko	2010	https://dx.doi.org/10.1111/j.1447-0756.2010.01203.x
	Zachar 2000	**Lack of protection against vertical transmission of HIV-1 by interferons produced during pregnancy in a cohort from East African republic of Malawi**	Zachar, V.; Fazio-Tirrozzo, G.; Fink, T.; Roberts, D. J.; Broadhead, R. L.; Brabin, B.; Ebbesen, P.	2000	
	Zhu 2014	**Maternal vitamin D status during pregnancy and bone mass in offspring at 20 years of age: a prospective cohort study**	Zhu, Kun; Whitehouse, Andrew J. O.; Hart, Prue H.; Kusel, Merci; Mountain, Jenny; Lye, Stephen; Pennell, Craig; Walsh, John P.	2014	https://dx.doi.org/10.1002/jbmr.2138
	Adank 2021	**Hypertensive Disorders of Pregnancy and Cognitive Impairment: A Prospective Cohort Study**	Adank, Maria C.; Hussainali, Rowina F.; Oosterveer, Lise C.; Ikram, M. Arfan; Steegers, Eric A. P.; Miller, Eliza C.; Schalekamp-Timmermans, Sarah	2021	https://dx.doi.org/10.1212/WNL.0000000000011363
	Brauner 2019	**The association between in-utero exposure to stressful life events during pregnancy and male reproductive function in a cohort of 20-year-old offspring: The Raine Study**	Brauner, E. V.; Hansen, A. M.; Doherty, D. A.; Dickinson, J. E.; Handelsman, D. J.; Hickey, M.; Skakkebaek, N. E.; Juul, A.; Hart, R.	2019	https://dx.doi.org/10.1093/humrep/dez070

Exclusion reason	Citation	Title	Authors	Published Year	DOI
	Looman 2018	**Pre-pregnancy dietary carbohydrate quantity and quality, and risk of developing gestational diabetes: the Australian Longitudinal Study on Women's Health**	Looman, Moniek; Schoenaker, Danielle A. J. M.; Soedamah-Muthu, Sabita S.; Geelen, Anouk; Feskens, Edith J. M.; Mishra, Gita D.	2018	https://dx.doi.org/10.1017/S0007114518001277
	Maugeri 2019	**Maternal Dietary Patterns Are Associated with Pre-Pregnancy Body Mass Index and Gestational Weight Gain: Results from the "Mamma & Bambino" Cohort**	Maugeri, Andrea; Barchitta, Martina; Favara, Giuliana; La Rosa, Maria Clara; La Mastra, Claudia; Magnano San Lio, Roberta; Agodi, Antonella	2019	https://dx.doi.org/10.3390/nu11061308
	Sivarajah 2018	**A 10 year retrospective cohort study: Pregnancy and neonatal outcomes in women with Sickle Cell disease at an East London Teaching Hospital**	Sivarajah, K.; Akhter, F.; Barroso, F.	2018	
	Zhao 2019	**Identifying appropriate pre-pregnancy body mass index classification to improve pregnancy outcomes in women of childbearing age in Beijing, China: a retrospective cohort study**	Zhao, Rui-Fen; Zhou, Li; Zhang, Wei-Yuan	2019	https://dx.doi.org/10.6133/apjcn.201909_28(3).0016
	Rankin 2010	**Maternal body mass index and congenital anomaly risk: a cohort study**	Rankin, J.; Tennant, P. W. G.; Stothard, K. J.; Bythell, M.; Summerbell, C. D.; Bell, R.	2010	https://dx.doi.org/10.1038/ijo.2010.66
	Zhang 2020	**Optimized cutoff maternal age for adverse obstetrical outcomes: a multicenter retrospective cohort study in Urban China during 2011 to 2012**	Zhang, Xiao-Lei; Liang, Huan; Zhao, Huan-Qiang; Wu, Su-Wen; Zhou, Qiong-Jie; Li, Xiao-Tian	2020	https://dx.doi.org/10.1097/CM9.0000000000000626
	vandeVijver 2010	**Pregnancy outcome after cervical conisation: a retrospective cohort study in the Leuven University Hospital**	van de Vijver, A.; Poppe, W.; Verguts, J.; Arbyn, M.	2010	https://dx.doi.org/10.1111/j.1471-0528.2009.02437.x

Exclusion reason	Citation	Title	Authors	Published Year	DOI
	Lundqvist 2016	**Vitamin D Status during Pregnancy: A Longitudinal Study in Swedish Women from Early Pregnancy to Seven Months Postpartum**	Lundqvist, Anette; Sandstrom, Herbert; Stenlund, Hans; Johansson, Ingegerd; Hultdin, Johan	2016	https://dx.doi.org/10.1371/journal.pone.0150385
	Mannava 2016	**Comparative Evaluation of C-reactive Proteins in Pregnant Women with and without Periodontal Pathologies: A Prospective Cohort Analysis**	Mannava, Padmakanth; Gokhale, Sunil; Pujari, Sudarshan; Biswas, Krishna P.; Kaliappan, Satish; Vijapure, Shashank	2016	
	Currie 2021	**Adverse childhood experiences are associated with illicit drug use among pregnant women with middle to high socioeconomic status: findings from the All Our Families Cohort**	Currie, Cheryl L.; Tough, Suzanne C.	2021	https://dx.doi.org/10.1186/s12884-021-03591-1
	Machado 2003	**Monitorização ambulatorial da pressão arterial em gestantes normotensas: estudo longitudinal da pressão arterial e da freqüência cardíaca materna nos três trimestres da gestação**	Machado, Antonio Vieira; Melo, Victor Hugo de; Nascimento Neto, Raimundo Marques	2003	
	Ramlau-Hansen 2008	**Semen quality according to prenatal coffee and present caffeine exposure: two decades of follow-up of a pregnancy cohort**	Ramlau-Hansen, C. H.; Thulstrup, A. M.; Bonde, J. P.; Olsen, J.; Bech, B. H.	2008	https://dx.doi.org/10.1093/humrep/den331
Retrospective pregnancy cohort (not enrolled during pregnancy)	Xue 2021	**Association of high maternal triglyceride levels early and late in pregnancy with adverse outcomes: A retrospective cohort study**	Xue, Rui-Hong; Wu, Dan-Dan; Zhou, Cheng-Liang; Chen, Lei; Li, Juan; Li, Zheng-Zheng; Fan, Jian-Xia; Liu, Xin-Mei; Lin, Xian-Hua; Huang, He-Feng	2021	https://dx.doi.org/10.1016/j.jacl.2020.10.001

Exclusion reason	Citation	Title	Authors	Published Year	DOI
	Voon 2017	**Comparison of midwife-led care and obstetrician-led care on maternal and neonatal outcomes in Singapore: A retrospective cohort study**	Voon, Shi Tian; Lay, Julie Tay Suan; San, Wilson Tam Wai; Shorey, Shefaly; Lin, Serena Koh Siew	2017	https://dx.doi.org/10.1016/j.midw.2017.07.010
	Moon 2020	**Safety of acupuncture during pregnancy: a retrospective cohort study in Korea**	Moon, H. Y.; Kim, M. R.; Hwang, D. S.; Jang, J. B.; Lee, J.; Shin, J. S.; Ha, I.; Lee, Y. J.	2020	https://dx.doi.org/10.1111/1471-0528.15925
	Londero 2019	**Maternal age and the risk of adverse pregnancy outcomes: a retrospective cohort study**	Londero, Ambrogio P.; Rossetti, Emma; Pittini, Carla; Cagnacci, Angelo; Driul, Lorenza	2019	https://dx.doi.org/10.1186/s12884-019-2400-x
	Londero 2009	**Couple risk factors for pregnancy: Related hypertensive disorders: A retrospective cohort study**	Londero, A. P.; Bertozzi, S.; Driul, L.; Marchesoni, D.	2009	http://dx.doi.org/10.1111/j.1479-828X.2009.01068.x
	Liu 2020	**Effect of pregravid obesity on perinatal outcomes in singleton pregnancies following in vitro fertilization and the weight-loss goals to reduce the risks of poor pregnancy outcomes: A retrospective cohort study**	Liu, Lu; Wang, Hongmei; Zhang, Yang; Niu, Jinlei; Li, Zhongyuan; Tang, Rong	2020	https://dx.doi.org/10.1371/journal.pone.0227766
	McInerney 2019	**Benefits of not smoking during pregnancy for Australian Aboriginal and Torres Strait Islander women and their babies: a retrospective cohort study using linked data**	McInerney, Carol; Ibiebele, Ibinabo; Ford, Jane B.; Randall, Deborah; Morris, Jonathan M.; Meharg, David; Mitchell, Jo; Milat, Andrew; Torvaldsen, Siranda	2019	https://dx.doi.org/10.1136/bmjopen-2019-032763
	Lawlor 2011	**Does maternal weight gain in pregnancy have long-term effects on offspring adiposity? A sibling study in a prospective cohort of 146,894 men from 136,050 families**	Lawlor, Debbie A.; Lichtenstein, Paul; Fraser, Abigail; Langstrom, Niklas	2011	https://dx.doi.org/10.3945/ajcn.110.009324

Exclusion reason	Citation	Title	Authors	Published Year	DOI
	CeaSoriano 2016	**Exposure to acid-suppressing drugs during pregnancy and the risk of asthma in childhood: an observational cohort study**	Cea Soriano, L.; Hernandez-Diaz, S.; Johansson, S.; Nagy, P.; Garcia-Rodriguez, L. A.	2016	https://dx.doi.org/10.1111/apt.13486
	Lee 2020	**Nationwide population-based cohort study of adverse obstetric outcomes in pregnancies with myoma or following myomectomy: retrospective cohort study**	Lee, Se Jin; Ko, Hyun Sun; Na, Sunghun; Bae, Jin Young; Seong, Won Joon; Kim, Jong Woon; Shin, Jaeeun; Cho, Hae Joong; Choi, Gyu Yeon; Kim, Jinsil; Cho, Geum Joon; Park, In Yang	2020	https://dx.doi.org/10.1186/s12884-020-03406-9
	Leybovitz-Haleluya 2018	**Maternal smoking during pregnancy and the risk of pediatric cardiovascular diseases of the offspring: A population-based cohort study with up to 18-years of follow up**	Leybovitz-Haleluya, Noa; Wainstock, Tamar; Landau, Daniella; Sheiner, Eyal	2018	https://dx.doi.org/10.1016/j.reprotox.2018.03.009
	Leybovitz-Haleluya 2018	**Maternal gestational diabetes mellitus and the risk of subsequent pediatric cardiovascular diseases of the offspring: a population-based cohort study with up to 18 years of follow up**	Leybovitz-Haleluya, Noa; Wainstock, Tamar; Landau, Daniella; Sheiner, Eyal	2018	https://dx.doi.org/10.1007/s00592-018-1176-1
	Lin 2019	**The gestational weight gain and perinatal outcomes among underweight women with twin pregnancies: Propensity score matched analysis from a three-year retrospective cohort**	Lin, Dongxin; Chen, Gengdong; Fan, Dazhi; Li, Pengsheng; Ma, Huiting; Wu, Shuzhen; Ye, Shaoxin; Rao, Jiaming; Zhou, Zixing; Liu, Yan; Guo, Xiaoling; Liu, Zhengping	2019	https://dx.doi.org/10.1016/j.ejogrb.2019.10.009

Exclusion reason	Citation	Title	Authors	Published Year	DOI
	Lin 2021	**Association between IVF/ICSI treatment and preterm birth and major perinatal outcomes among dichorionic-diamnionic twin pregnancies: A seven-year retrospective cohort study**	Lin, Dongxin; Li, Pengsheng; Fan, Dazhi; Chen, Gengdong; Wu, Shuzhen; Ye, Shaoxin; Ma, Huiting; Rao, Jiaming; Zhou, Zixing; Zeng, Meng; Huang, Zheng; Guo, Xiaoling; Liu, Zhengping	2021	https://dx.doi.org/10.1111/aogs.13981
	Liu 2019	**The risk of preterm delivery and pregnancy outcomes in women with asymptomatic short cervix: a retrospective cohort study**	Liu, Cathy Z.; Ho, Nicole; Nguyen, Anh D.; Lehner, Christoph; Sekar, Renuka; Amoako, Akwasi A.	2019	https://dx.doi.org/10.1080/14767058.2019.1647163
	Rodrigues 2019	**The impact of different classes of lupus nephritis on maternal and fetal outcomes: a cohort study of 147 pregnancies**	Rodrigues, B. Costa; Lacerda, M. Ignacchiti; Ramires de Jesus, G. R.; Cunha Dos Santos, Flavia; Ramires de Jesus, N.; Levy, R. A.; Klumb, E. Mendes	2019	https://dx.doi.org/10.1177/0961203319829825
	Tsai 2018	**Maternal folic acid supplementation during pregnancy in relation to childhood rhinitis: A cohort study in Taiwan**	Tsai, Z.; Huang, Y.; Huang, Y.; Lin, C.; Fang, H.; Chiu, C.; Kao, P.; Weng, T.; Lu, K.; Lee, W.; Gau, C.; Tsai, H.; Yao, T.	2018	
	Wang 2014	**Independent and joint effects of prenatal maternal smoking and maternal exposure to second-hand smoke on the development of adolescent obesity: a longitudinal study**	Wang, Liang; Mamudu, Hadii M.; Alamian, Arsham; Anderson, James L.; Brooks, Billy	2014	https://dx.doi.org/10.1111/jpc.12667
	Wittmaack 2019	**Maternal History of Cervical Surgery and Preterm Delivery: A Retrospective Cohort Study**	Wittmaack, Amaya; Dudley, Donald; Boyle, Annelee	2019	https://dx.doi.org/10.1089/jwh.2018.7457

Exclusion reason	Citation	Title	Authors	Published Year	DOI
	Linet 2018	**Prospective investigation of folic acid supplements before and during early pregnancy and paediatric and adult cancers in the Chinese children and families cohort: a pilot study in a sample of rural and urban families**	Linet, Martha S.; Wang, Linhong; Wang, Ning; Berry, Robert J.; Chao, Ann; Hao, Ling; Li, Zhu; Fang, Liwen; Yin, Peng; Potischman, Nancy; Sun, Xin; Meng, Fanweng; Yang, Ruilan; Cong, Shu; Fan, Jing; Kitahara, Cari M.; Liang, Xiaofeng; Liu, Fang; Lu, Xiaojun; Lv, Fan; Mu, Chunhua; Sampson, Joshua; Tang, Yongmin; Wan, Weiqing; Wang, Baohua; Wang, Hongsheng; Zhang, Leping; Wang, Yu	2018	https://dx.doi.org/10.1136/bmjopen-2018-022394
Wrong study design (data exclusively from national registries etc., cohort not limited to pregnant women, RCT)	Batty 2006	**Effect of maternal smoking during pregnancy on offspring's cognitive ability: empirical evidence for complete confounding in the US national longitudinal survey of youth**	Batty, G. David; Der, Geoff; Deary, Ian J.	2006	
	vanderWaerden 2015	**Predictors of persistent maternal depression trajectories in early childhood: results from the EDEN mother-child cohort study in France**	van der Waerden, J.; Galera, C.; Saurel-Cubizolles, M. J.; Sutter-Dallay, A. L.; Melchior, M.; Eden Mother-Child Cohort Study Group	2015	https://dx.doi.org/10.1017/S003329171500015X
	Andersen 2012	**Use of prescription paracetamol during pregnancy and risk of asthma in children: a population-based Danish cohort study**	Andersen, Ane Birgitte Telen; Farkas, Dora Kormendine; Mehnert, Frank; Ehrenstein, Vera; Erichsen, Rune	2012	https://dx.doi.org/10.2147/CLEP.S28312

Exclusion reason	Citation	Title	Authors	Published Year	DOI
	Andersen 2016	**Re: Attention deficit hyperactivity disorder and autism spectrum disorder in children born to mothers with thyroid dysfunction: a Danish nationwide cohort study Maternal hypothyroidism and risk of autism Reply**	Andersen, S. L.; Laurberg, P.	2016	10.1111/1471-0528.13954
	Artama 2013	**Effects of maternal epilepsy and antiepileptic drug use during pregnancy on perinatal health in offspring: nationwide, retrospective cohort study in Finland**	Artama, Miia; Gissler, Mika; Malm, Heli; Ritvanen, Annukka; Drug,; Pregnancy, Group	2013	https://dx.doi.org/10.1007/s40264-013-0052-8
	Beau 2016	**Atropinic burden of drugs during pregnancy and psychological development of children: a cohort study in the EFEMERIS database**	Beau, Anna-Belle; Montastruc, Jean-Louis; Lacroix, Isabelle; Montastruc, Francois; Hurault-Delarue, Caroline; Damase-Michel, Christine	2016	https://dx.doi.org/10.1111/bcp.12978
	Bin 2017	**IMPACT OF MATERNAL SLEEP APNOEA ON CHILDHOOD HEALTH AND DEVELOPMENTAL OUTCOMES: LONGITUDINAL POPULATION RECORD LINKAGE STUDY**	Bin, Y.; Roberts, C.; Cistulli, P.; Ford, J.	2017	10.1111/jsr.52_12618
	Blomstrom 2016	**Associations Between Maternal Infection During Pregnancy, Childhood Infections, and the Risk of Subsequent Psychotic Disorder--A Swedish Cohort Study of Nearly 2 Million Individuals**	Blomstrom, Asa; Karlsson, Hakan; Gardner, Renee; Jorgensen, Lena; Magnusson, Cecilia; Dalman, Christina	2016	https://dx.doi.org/10.1093/schbul/sbv112
	Braback 2018	**Childhood asthma and smoking exposures before conception-A three-generational cohort study**	Braback, Lennart; Lodge, Caroline J.; Lowe, Adrian J.; Dharmage, Shyamali C.; Olsson, David; Forsberg, Bertil	2018	https://dx.doi.org/10.1111/pai.12883

Exclusion reason	Citation	Title	Authors	Published Year	DOI
	Byrjalsen 2013	**Use of Corticosteroids during Pregnancy and in the Postnatal Period and Risk of Asthma in Offspring: A Nationwide Danish Cohort Study**	Byrjalsen, A.; Froeslev, T.; Andersen, A. B. T.; Olsen, M.; Soerensen, H. T.	2013	
	Checkley 2009	**EFFECTS OF MATERNAL VITAMIN A SUPPLEMENTATION ON LUNG FUNCTION IN PREADOLESCENT OFFSPRING: A FOLLOW-UP STUDY OF A RANDOMIZED, DOUBLE-BLINDED, PLACEBO-CONTROLLED TRIAL COHORT IN RURAL NEPAL**	Checkley, W.; West, K. P.; Wise, R.; Baldwin, M. R.; LeClerq, S. C.; Wu, L.; Christian, P.; Katz, J.; Tielsch, J.; Khatry, S.; Sommer, A.	2009	
	Chen 2013	**Maternal pre-pregnancy BMI and offspring ADHD: A population based cohort study using a sibling-comparison design**	Chen, Q.; Sjolander, A.; Langstrom, N.; Rodriguez, A.; Serlachius, E.; D'Onofrio, B. M.; Lichtenstein, P.; Larsson, H.	2013	http://dx.doi.org/10.1007/s10654-013-9820-0
	Fan 2020	**Associations between macrolide antibiotics prescribing during pregnancy and adverse child outcomes in the UK: population based cohort study (vol 368, m331, 2020)**	Fan,	2020	10.1136/bmj.m766

Exclusion reason	Citation	Title	Authors	Published Year	DOI
	Felix 2018	**Cohort Profile: Pregnancy And Childhood Epigenetics (PACE) Consortium**	Felix, Janine F.; Joubert, Bonnie R.; Baccarelli, Andrea A.; Sharp, Gemma C.; Almqvist, Catarina; Annesi-Maesano, Isabella; Arshad, Hasan; Baiz, Nour; Bakermans-Kranenburg, Marian J.; Bakulski, Kelly M.; Binder, Elisabeth B.; Bouchard, Luigi; Breton, Carrie V.; Brunekreef, Bert; Brunst, Kelly J.; Burchard, Esteban G.; Bustamante, Mariona; Chatzi, Leda; Cheng Munthe-Kaas, Monica; Corpeleijn, Eva; Czamara, Darina; Dabelea, Dana; Davey Smith, George; De Boever, Patrick; Duijts, Liesbeth; Dwyer, Terence; Eng, Celeste; Eskenazi, Brenda; Everson, Todd M.; Falahi, Fahimeh; Fallin, M. Daniele; Farchi, Sara; Fernandez, Mariana F.; Gao, Lu; Gaunt, Tom R.; Ghantous, Akram; Gillman, Matthew W.; Gonseth, Semira; Grote, Veit; Gruzieva, Olena; Haberg, Siri E.; Herceg, Zdenko; Hivert, Marie-France; Holland, Nina; Holloway, John W.; Hoyo, Cathrine; Hu, Donglei; Huang, Rae-Chi; Huen, Karen; Jarvelin, Marjo-Riitta; Jima, Dereje D.; Just, Allan C.; Karagas, Margaret R.; Karlsson, Robert; Karmaus, Wilfried; Kechris, Katerina J.; Kere, Juha; Kogevinas, Manolis; Koletzko, Berthold; Koppelman, Gerard H.; Kupers, Leanne K.; Ladd-Acosta, Christine; Lahti, Jari; Lambrechts, Nathalie; Langie, Sabine A. S.; Lie, Rolv T.; Liu, Andrew H.; Magnus, Maria C.; Magnus, Per; Maguire, Rachel L.; Marsit, Carmen J.; McArdle, Wendy; Melen, Erik; Melton, Phillip; Murphy, Susan K.; Nawrot, Tim S.; Nistico, Lorenza; Nohr, Ellen A.; Nordlund, Bjorn; Nystad, Wenche; Oh, Sam S.; Oken, Emily; Page, Christian M.; Perron, Patrice; Pershagen, Goran; Pizzi, Costanza; Plusquin, Michelle; Raikkonen, Katri; Reese, Sarah E.; Reischl, Eva; Richiardi, Lorenzo; Ring, Susan; Roy, Ritu P.; Rzehak, Peter; Schoeters, Greet; Schwartz, David A.; Sebert, Sylvain;	2018	https://dx.doi.org/10.1093/ije/dyx190

Exclusion reason	Citation	Title	Authors	Published Year	DOI
			Snieder, Harold; Sorensen, Thorkild I. A.; Starling, Anne P.; Sunyer, Jordi; Taylor, Jack A.; Tiemeier, Henning; Ullemar, Vilhelmina; Vafeiadi, Marina; Van Ijzendoorn, Marinus H.; Vonk, Judith M.; Vriens, Annette; Vrijheid, Martine; Wang, Pei; Wiemels, Joseph L.; Wilcox, Allen J.; Wright, Rosalind J.; Xu, Cheng-Jian; Xu, Zongli; Yang, Ivana V.; Yousefi, Paul; Zhang, Hongmei; Zhang, Weiming; Zhao, Shanshan; Agha, Golareh; Relton, Caroline L.; Jaddoe, Vincent W. V.; London, Stephanie J.		

Exclusion reason	Citation	Title	Authors	Published Year	DOI
	Freud 2018	**Maternal chorioamnionitis & long term neurological morbidity in the offspring - results from a population based cohort analysis**	Freud, A.; Sheiner, E.; Wainstock, T.; Beloosesky, R.; Fischer, L.; Landau, D.; Walfisch, A.	2018	
	Hagberg 2017	**Maternal Depression and Antidepressant Use During Pregnancy and Risk of Autism Spectrum Disorders in Offspring: Population-based Cohort and Bidirectional Case-Crossover Sibling Study**	Hagberg, K. W.; Robijn, A.; Jick, S. S.	2017	
	Haleluya 2018	**Maternal smoking during pregnancy and the risk of pediatric cardiovascular diseases of the offspring: A population-based cohort study with up to 18-years follow up**	Haleluya, N. L.; Wainstoc, T.; Sheiner, E.	2018	
	Hargreave 2014	**Risk of cancer in children born after maternal use of fertility drugs: results from a nationwide Danish population-based cohort study**	Hargreave, M.; Jensen, A.; Nielsen, T. S. S.; Colov, E. P.; Andersen, K. K.; Pinborg, A.; Kjaer, S. K.	2014	
	Hargreave 2017	**Maternal use of Hormonal Contraceptives and Risk of Childhood Leukaemia - A Nationwide Population-Based Cohort Study**	Hargreave, M.; Morch, L. S.; Winther, J. F.; Schmiegelow, K.; Kjaer, S. K.	2017	
	Havdahl 2019	**POLYGENIC RISK FOR ADHD AND EXPOSURES DURING PREGNANCY IN THE NORWEGIAN MOTHER AND CHILD COHORT STUDY (MOBA)**	Havdahl, A.; Hannigan, L.; Askeland, R. B.; Ask, H.; Tesli, M.; Corfield, E.; Oyen, A. S.; Andreassen, O.; Smith, G. D.; Reichborn-Kjennerud, T.	2019	10.1016/j.euroneuro.2019.07.216
	Jordan 2020	**Introduction to the Special Issue on "Informing Longitudinal Studies on the Effects of Maternal Stress and Substance Use on Child Development: Planning for**	Jordan, Chloe J.; Weiss, Susan R. B.; Howlett, Katia D.; Freund, Michelle P.	2020	https://dx.doi.org/10.1007/s42844-020-00022-6

Exclusion reason	Citation	Title	Authors	Published Year	DOI
		**the HEALthy Brain and Child Development (HBCD) Study"**			
	Lee 2015	**Maternal Use of Prenatal Nutritional Supplements and Risk of Autism in the Stockholm Youth Cohort**	Lee, B.; DeVilbiss, E. A.; Magnusson, C.	2015	10.1016/s0924-9338(15)30184-x
	Leybovitz-Haleluya 2019	**Maternal preeclampsia and the risk of gastrointestinal disease of the offspring: A population-based cohort study**	Leybovitz-Haleluya, N. S.; Wainstock, T.; Sheiner, E.	2019	10.1016/j.ajog.2018.11.744
	Li 2011	**PRENATAL STRESS FOLLOWING MATERNAL BEREAVEMENT DURING PREGNANCY AND OVERWEIGHT IN THE OFFSPRING: A COHORT STUDY IN 109 919 DANISH CONSCRIPTS**	Li, J.; Obel, C.; Vestergaard, M.; Olsen, J.	2011	10.1136/jech.2011.142976i.88
	Munger 2017	**Maternal levels of Epstein-Barr virus IgG antibodies and risk of multiple sclerosis in offspring in the Finnish Maternity Cohort**	Munger, K.; Zhang, Z. L.; Aivo, J.; Hongell, K.; Soilu-Hanninen, M.; Surcel, H. M.; Ascherio, A.	2017	
	Nielsen 2010	**Maternal blood glucose in diabetic pregnancies and cognitive performance in offspring in young adulthood: a Danish cohort study**	Nielsen, G. L.; Andersen, E.; Lundbye-Christensen, S.	2010	https://dx.doi.org/10.1111/j.1464-5491.2010.03024.x
	Perng 2020	**In utero exposure to gestational diabetes mellitus and cardiovascular risk factors in youth: A longitudinal analysis in the EPOCH cohort**	Perng, W.; Hockett, C. W.; Sauder, K. A.; Dabelea, D.	2020	10.1111/ijpo.12611
	Plana-Ripoll 2016	**Maternal stress before and during pregnancy and subsequent infertility in daughters: a nationwide population-based cohort study**	Plana-Ripoll, O.; Li, J.; Kesmodel, U. S.; Olsen, J.; Parner, E.; Basso, O.	2016	https://dx.doi.org/10.1093/humrep/dev309

Exclusion reason	Citation	Title	Authors	Published Year	DOI
	Plana-Ripoll 2015	**Prenatal Maternal Bereavement and Adult Reproductive Impairments: A Population-Based Cohort Study**	Plana-Ripoll, O.; Olsen, J.; Li, J.	2015	
	Singal 2016	**Neonatal and childhood neurodevelopmental, health and educational outcomes of children exposed to antidepressants and maternal depression during pregnancy: protocol for a retrospective population-based cohort study using linked administrative data**	Singal, Deepa; Brownell, Marni; Chateau, Dan; Ruth, Chelsea; Katz, Laurence Y.	2016	https://dx.doi.org/10.1136/bmjopen-2016-013293
	Stokholm 2014	**Maternal propensity for infections and risk of childhood asthma: a registry-based cohort study**	Stokholm, Jakob; Sevelsted, Astrid; Bonnelykke, Klaus; Bisgaard, Hans	2014	https://dx.doi.org/10.1016/S2213-2600(14)70152-3
	Straatmann 2018	**ALCOHOL CONSUMPTION IN PREGNANCY AND CHILDHOOD HEARING AND NEURODEVELOPMENTAL PROBLEMS IN THE UK: ANALYSIS FROM THE MILLENNIUM COHORT STUDY**	Straatmann, V. S.; Taylor-Robinson, D. C.; Bergh, E. H.; Fleming, K. M.	2018	10.1136/jech-2018-SSMabstracts.54
	Taylor 2019	**Change in maternal smoking behaviour between the first two singleton live pregnancies and childhood obesity: analysis of a UK population-based cohort**	Taylor, E. J.; Ziauddeen, N.; Godfrey, K. M.; Berrington, A.; Alwan, N. A.	2019	
	Virk 2014	**In-utero exposure to bereavement and offspring IQ: a Danish national cohort study**	Virk, Jasveer; Obel, Carsten; Li, Jiong; Olsen, Jorn	2014	https://dx.doi.org/10.1371/journal.pone.0088477
	Wainstock 2018	**Maternal obesity and offspring pediatric cardiovascular morbidity- Results from a population based cohort study**	Wainstock, T.; Walfisch, A.; Landau, D.; Sheiner, E.	2018	
	Wainstock 2018	**Prenatal maternal smoking and offspring respiratory morbidity: Results of a population based cohort study**	Wainstock, T.; Walfisch, A.; Sheiner, E.	2018	

Exclusion reason	Citation	Title	Authors	Published Year	DOI
	Wainstock 2018	**Maternal anemia and pediatric neurological morbidity in the offspring - results from a population based cohort analysis**	Wainstock, T.; Walfisch, A.; Sheiner, E.	2018	
	Yeates 2013	**Fish consumption and polyunsaturated fatty acid status of pregnant women in the Seychelles Child Development and Nutrition Study Cohort 2**	Yeates, A. J.; Mulhern, M. S.; McSorley, E. M.; Wallace, J. M. W.; Watson, G. E.; Myers, G. J.; van Wijngaarden, E.; Shamlaye, C.; Henderson, J.; Thurston, S. W.; Davidson, P. W.; Strain, J. J.	2013	10.1017/s0029665113001183
	Meeraus 2015	**Association between antibiotic prescribing in pregnancy and cerebral palsy or epilepsy in children born at term: a cohort study using the health improvement network**	Meeraus, Wilhelmine Hadler; Petersen, Irene; Gilbert, Ruth	2015	https://dx.doi.org/10.1371/journal.pone.0122034
	Kumordzie 2020	**Maternal and child factors associated with child body fatness in a Ghanaian cohort**	Kumordzie, Sika M.; Okronipa, Harriet; Arimond, Mary; Adu-Afarwuah, Seth; Ocansey, Maku E.; Young, Rebecca R.; Bentil, Helena J.; Tamakloe, Solace M.; Oaks, Brietta M.; Dewey, Kathryn G.	2020	https://dx.doi.org/10.1017/S1368980019001745
	AbuFreha 2020	**Maternal Hepatitis B Virus or Hepatitis C Virus Carrier Status and Long-Term Endocrine Morbidity of the Offspring-A Population-Based Cohort Study**	Abu Freha, Naim; Wainstock, Tamar; Menachem, Tzvi Najman; Sheiner, Eyal	2020	https://dx.doi.org/10.3390/jcm9030796

Exclusion reason	Citation	Title	Authors	Published Year	DOI
	Liu 2018	**Maternal pregestational or gestational diabetes and childhood wheezing: A population-based cohort study**	Liu, Xiaoqin; Agerbo, Esben; Li, Jiong; Dharmage, Shyamali C.; Thomsen, Reimar W.; Olsen, Jorn; Munk-Olsen, Trine	2018	https://dx.doi.org/10.1111/all.13551
	Cole 2009	**(Young investigator award) Maternal diet during pregnancy and childhood bonemass ataged 9 years: A longitudinal study**	Cole, Z.; Gale, C.; Javaid, K.; Robinson, S.; Law, C.; Boucher, B.; Crozier, S.; Godfrey, K.; Dennison, E.; Cooper, C.	2009	http://dx.doi.org/10.1007/s00198-009-0923-0
	Anonymous 2018	**Corrigendum: Cohort Profile: Pregnancy And Childhood Epigenetics (PACE) Consortium [Int J Epidemiol., (2017)] DOI 10.1093/ije/dyx190**	Anonymous,	2018	http://dx.doi.org/10.1093/ije/dyx220
	Qu 2020	**Effect of the Interaction Between Pre-pregnancy Body Mass Index and Fresh/Frozen Embryo Transfer on Perinatal Outcomes of Assisted Reproductive Technology-Conceived Singletons: A Retrospective Cohort Study**	Qu, Pengfei; Mi, Yang; Zhao, Doudou; Wang, Min; Dang, Shaonong; Shi, Wenhao; Shi, Juanzi	2020	https://dx.doi.org/10.3389/fendo.2020.560103
	Suresh 2011	**Lung Function At The Age Of 21 Years: Pregnancy And Birth Related Factors; Findings From A Longitudinal Cohort Study**	Suresh, S.; Sly, P.; O'Callaghan, M.; Mamun, A. A.	2011	
	Wahlbeck 2000	**Low maternal body mass index, small size at birth and thinness during childhood are associated with schizophrenia: A population based cohort study**	Wahlbeck, K.; Forsen, T.; Osmond, C.; Barker, D. J. P.; Eriksson, J. G.	2000	10.1016/s0920-9964(00)90462-6
	Werler 2015	**Bias from conditioning on live-births in pregnancy cohorts: an illustration based on neurodevelopment in children after prenatal exposure to organic pollutants (Liew et al. 2015)**	Werler, Martha M.; Parker, Samantha E.	2015	https://dx.doi.org/10.1093/ije/dyv139

Exclusion reason	Citation	Title	Authors	Published Year	DOI
	Smith 2019	**Associations between early maternal behaviours and child language at 36 months in a cohort experiencing adversity**	Smith, Jodie; Levickis, Penny; Eadie, Tricia; Bretherton, Lesley; Conway, Laura; Goldfeld, Sharon	2019	https://dx.doi.org/10.1111/1460-6984.12435
	Young 2018	**Role of maternal preconception nutrition on offspring growth and risk of stunting across the first 1000 days in Vietnam: A prospective cohort study**	Young, Melissa F.; Nguyen, Phuong Hong; Gonzalez Casanova, Ines; Addo, O. Yaw; Tran, Lan Mai; Nguyen, Son; Martorell, Reynaldo; Ramakrishnan, Usha	2018	https://dx.doi.org/10.1371/journal.pone.0203201
	Leon-Aguilar 2019	**Maternal obesity leads to long-term altered levels of plasma ceramides in the offspring as revealed by a longitudinal lipidomic study in children**	Leon-Aguilar, Luis Felipe; Croyal, Mikael; Ferchaud-Roucher, Veronique; Huang, Fengyang; Marchat, Laurence A.; Barraza-Villarreal, Albino; Romieu, Isabelle; Ramakrishnan, Usha; Krempf, Michel; Ouguerram, Khadija; Mercado-Camargo, Rosalio; Bolanos-Jimenez, Francisco	2019	https://dx.doi.org/10.1038/s41366-018-0291-y
	Smith 2020	**Association between vitamin D status in early pregnancy and atopy in offspring in a vitamin D deplete cohort**	Smith, Maeve; O'Brien, Eileen C.; Alberdi, Goiuri; Geraghty, Aisling A.; Kilbane, Mark; McKenna, Malachi J.; McAuliffe, Fionnuala M.	2020	https://dx.doi.org/10.1007/s11845-019-02078-5
	Eskenazi 2009	**Associations Between Maternal PBDE Serum Concentrations and Child**	Eskenazi, B.; Marks, A.; Chevrier, J.; Harley, K.; Bradman, A.; Sjodin, A.	2009	10.1097/01.ede.0000362995.59556.bf

Exclusion reason	Citation	Title	Authors	Published Year	DOI
		**Neurodevelopment in the Chamacos Cohort**			

Additional File 4: Data extraction form

General information: Ongoing and completed pregnancy and prepregnancy cohorts

Year of publication

Last name of first author

Title of publication

Title of paper / abstract / report that data are extracted from

Overarching aim of cohort study

Pull this from the abstract

Full name of corresponding author

Full cohort name

The cohort name can be the same as the study name. If neither a cohort name or a study name is listed, write "not available"

Cohort acronym

If any

Does the cohort have sites in > 1 country

1. Yes
2. NO

Clear above selection

Cohort region

1. North America
2. Central & South America
3. Europe
4. Asia
5. Africa
6. Oceana

Clear above selection

Cohort location (country, city)

First year of cohort study recruitment

Last year of cohort study recruitment

This could be the same as the first year

Last year that cohort followed up with infants or children

Please mention as Not specified if not mentioned

Type of cohort

1. Pregnancy cohort
2. Pre-pregnancy cohort
3. Both pregnancy & pre-pregnancy cohort
4. Unsure

Clear above selection

Is this a multisite (> 1 site) cohort?

This could be that the cohort takes place in >1 hospital or >1 city or >1 country

1. Yes, multisite cohort
2. No, single site cohort
3. Unsure

Clear above selection

Copy the text from manuscript on type of cohort

# (N) of pregnant women included in cohort

Copy from manuscript

# (N) of children included in cohort

Copy from manuscript

Cohort inclusion criteria (one sentence summary max)

Please do write a summary of 2-3 sentences

Cohort exclusion criteria (one sentence summary max)

Copy manuscript text for cohort inclusion/exclusion criteria

What is the source population for the cohort?

by geography or service delivery or other, but NOT by time

1. maternity ward of hospital or group of hospitals (identified through where they give birth + geography)
2. prenatal or antenatal service recipient + geography
3. hospital or group of hospitals catchement area (not linked to receipt of prenatal care or maternity care)
4. geographic region ONLY (not associated with a certain hospital or hospital system)
5. Other

Clear above selection

How were women identified for participation from the source population?

1. All eligible women were recruited
2. A random sample of women that met inclusion criteria were recruited
3. Other

Clear above selection

How does recruitment relate to time?

e.g., only women who delivered between XX and XX dates are included. Only women born in a certain year were included

Copy text from article related to source population & recruitmetn here.

Copy text from the study. This could be recruitment at prenatal care visits, recruitment of all women in a certain town, etc.

Duration of follow-up of infants & children

Until what age are they followed? Specify whether this is months or years

Frequency of follow-up of infants & children (input specific ages when follow-up is scheduled to happen)

Copy from manuscript

Follow-up criteria (if any) for infants & children (if none, write no follow-up critieria specified)

Please write summary of 1-2 sentences

Copy manuscript text on follow-up criteria

How was the cohort study funded?

Do the authors mention whether the informed consent included broad consent for future use of data or samples?

Look for words like "data reuse" "broad consent" "data sharing" "future use" "biorepository" "biobank" "tissue bank"

1. Yes
2. No
3. Unsure

Clear above selection

Copy manuscript text related to broad consent for future use of data or samples

Maternal exposures measured by the cohort

Was maternal nutrition measured by cohort?

1. Yes
2. No

Clear above selection

Was gestational diabetes measured by the cohort?

1. Yes
2. No

Clear above selection

Was preeclampsia measured by the cohort?

1. Yes
2. No

Clear above selection

Was maternal C reactive protein measured by the cohort?

1. Yes
2. No

Clear above selection

Was maternal T cell count (or other immune response mesure) measured by the cohort?

1. Yes
2. No

Clear above selection

Was maternal vaccination history, including vaccinations received during pregnancy, measured by the cohort?

1. Yes
2. No

Clear above selection

Was maternal medication measured?

1. Yes
2. No

Clear above selection

Was maternal consumption of illicit drugs measured for the cohort?

1. Yes
2. No

Clear above selection

Was maternal exposure to STORCH pathogens measured?

STORCH=syphilis, toxoplasmosis, other infections, rubella, cytomegalovirus infection and herpes.

1. Yes
2. No

Clear above selection

Which STORCH pathogens were measured in the pregnat women?

TORCH infections are a group of congenital infections that are passed from mother to child at some time during pregnancy, during delivery, or after birth.

STORCH=syphilis, toxoplasmosis, other infections, rubella, cytomegalovirus infection and herpes.

Was maternal HIV status measured?

HIV=human immunodeficiency virus

1. Yes
2. No

Clear above selection

Was maternal NTDs (neglected tropical diseases) status measured?

NCD= Noncummunicable diseases; NTD=neural tube defect

1. Yes
2. No

Clear above selection

Which maternal NTDs were measured? (e.g. malaria, Zika virus, Dengue, etc)

Was maternal NCDs (non communicable diseases) status measured?

1. Yes
2. No

Clear above selection

Which NCDs were measured for the pregnant woman? (e.g. cancer, heart disease, diabetes, etc.)

Was maternal microbiome assessed? (as with stool sample)

The microbiome comprises all of the genetic material within a microbiota (the entire collection of microorganisms in a specific niche, such as the human gut)

1. Yes
2. No

Clear above selection

Was maternal metabolomics measured? (as through nuclear magnetic resonance (NMR) or mass spectrometry (MS) or gas chromatography–mass spectrometry (GC-MS))

Metabolomics is the large-scale study of small molecules, commonly known as metabolites, within cells, biofluids, tissues or organisms. Collectively, these small molecules and their interactions within a biological system are known as the metabolome.

1. Yes
2. No

Clear above selection

Was maternal immune function assessed? (as through T cell count)

1. Yes
2. No

Clear above selection

Were maternal socio-demographic factors measured?

1. Yes
2. No

Clear above selection

Which socio-demographics factors were measured?

For example: income, education, insurance status, etc

Was maternal race/minority status measured?

1. Yes
2. No

Clear above selection

What categories were included for race/minority status (e.g. ethnic or racial minority, religous minority, migrant or refugee)?

Was maternal disability measured?

1. Yes
2. No

Clear above selection

Was maternal exposure to IPV during or prior to pregnancy measured?

1. Yes

2. No

Clear above selection

What tools or questions were used to measure exposure to IPV?

Was maternal depression measured?

1. Yes
2. No

Clear above selection

What tool was used to measure maternal depression (e.g. CESD)?

List names and abbreviations of all despression screening tools

Was maternal exposure to occupational hazards measured?

1. Yes
2. No

Clear above selection

Was maternal exposure to indoor air quality measured?

1. Yes
2. No

Clear above selection

Was maternal exposure to environmental toxins measured?

For example: lead;

mercury;radon; benzene, etc

1. Yes
2. No

Clear above selection

List additional maternal exposures that were measured, but not mentioned above.

Copy the text from the manuscript. Mention any pre-pregnancy exposures here.

Infant and child outcomes measured by the cohort

Was infant head circumference measured for the infant or child?

1. Yes
2. No

Clear above selection

Which infant anthropometric measures (other than head circumference) were recorded?

Anthropometric measures commonly used as indices of growth and development for infants include length, weight, and head circumference.

Was the infant or child growth curve assessed?

1. Yes
2. No

Clear above selection

Was nutrition status measured for the infant/child?

1. Yes
2. No

Clear above selection

Was immune function measured for the infant or child? (as through T cell count)

1. Yes
2. No

Clear above selection

Was C reactive protein level measured for the infant or child?

1. Yes
2. No

Clear above selection

Was medication use measured for the infant or child?

1. Yes
2. No

Clear above selection

Was under 5 mortality measured?

1. Yes
2. No

Clear above selection

Was microbiome assessed for the infant or child? (as through stool sample)

The microbiome comprises all of the genetic material within a microbiota (the entire collection of microorganisms in a specific niche, such as the human gut)

1. Yes
2. No

Clear above selection

Was metabolomics measured for the infant or child? (as through nuclear magnetic resonance (NMR) or mass spectrometry (MS), or gas chromatography–mass spectrometry (GC-MS))

Metabolomics is the large-scale study of small molecules, commonly known as metabolites, within cells, biofluids, tissues or organisms. Collectively, these small molecules and their interactions within a biological system are known as the metabolome.

1. Yes
2. No

Clear above selection

Was cognitive development measured for the infant or child?

1. Yes
2. No

Clear above selection

List the tools that were used to measure cognitive development and the ages at which they were applied

	Tool 1 name	Tool 2 name	Tool 3 name	Tool 4 name	Tool 5 name	Tool 6 name
**Tool name**	column Tool 1 name row Tool name	column Tool 2 name row Tool name	column Tool 3 name row Tool name	column Tool 4 name row Tool name	column Tool 5 name row Tool name	column Tool 6 name row Tool name
**Ages applied**	column Tool 1 name row Ages applied	column Tool 2 name row Ages applied	column Tool 3 name row Ages applied	column Tool 4 name row Ages applied	column Tool 5 name row Ages applied	column Tool 6 name row Ages applied

Were child educational outcomes measured?

1. Yes
2. No

Clear above selection

Were child economic outcomes measured?

1. Yes
2. No

Clear above selection

Was infant or child exposure to violence measured?

1. Yes
2. No

Clear above selection

What tools or questions were used to measure child exposure to violence?

Write out the full name of the tool if they mention a tool

What other infant or child outcomes were measured?

Copy the text from the manuscript. If not measured, then mention NA

Sample collection and availability in pregnancy and pre-pregnancy cohorts

Maternal samples collected prior to pregnancy

What samples were collected from the woman prior to pregnancy (e.g. urine, blood, fecal, saliva, etc)?

How often were samples collected from the woman prior to pregnancy?

Maternal samples collected during pregnancy

What samples were collected from the pregnant woman during pregnancy? (e.g. vaginal swab, urine, blood, fecal, saliva, etc)

At what gestational ages were samples collected from the pregnant woman?

If at enrollment and at birth, say that here.

Fetal samples

What samples were collected for the fetus or placenta? (e.g. urine, blood, fecal, saliva, etc)

For example: Blood, Urine, Vaginal swab

At what gestational ages were the fetal or placental samples collected?

If at enrollment and at birth, say that here.

Paternal samples collected during pregnancy

What samples were collected from the biological father? (e.g. semen, urine, blood, fecal, saliva, etc)

What is the frequency of collection of samples from the biological father?

If at enrollment and at birth, say that here.

Infant samples

What samples were collected from the infant? (e.g. urine, blood, fecal, saliva, etc)

At what ages were the samples collected from the infant?

Child samples

What samples were collected from the child? (e.g. urine, blood, fecal, saliva, etc)

At what ages were the samples collected from the child?

Sample storage in a biorepository or tissue bank

Do the authors mention storing the samples in a biorepository, biobank, or tissue bank?

1. Yes
2. No
3. Unsure

Clear above selection

What is the name of the biorepository, biobank, or tissue bank?

Copy text from manuscript

Where is the biorepository, biobank, or tissue bank located?

Do the authors mention whether the samples can be accessed for additional research?

1. Yes
2. No
3. Unsure

Clear above selection

How can samples be accessed?

Copy text from manuscript, look online if a biorepository is mentioned, but the infromation on access is not provided.

Provide a link to description of how to access samples through biorepository, biobank, or tissue bank.

What is the source of funding for sample storage?

Copy text from manuscript. If none mentioned, write "None mentioned"

Overview of pregnancy and pre-pregnancy cohort registries and data sharing platforms

Do the authors mention sharing participant level data to a platform, registry, dataverse or other?

Look for words like "data availability" "data stored" "data access" "dataverse"

1. Yes

2. No
3. Unsure

Clear above selection

If yes, what is the registry or platform name where the data can be accessed

If yes, what is the website for the registry or platform where data can be accessed?

Do the authors mention including cohort metadata (study level data) in a cohort browser or other type of metadata registry?

Look for words like "cohort browser" or "metadata registry"

1. Yes
2. No
3. Unsure

Clear above selection

If yes, what is the cohort browser or metadata registry name?

If yes, what is the website for the cohort browser or metadata registry?

Additional File 5: Cohort aims

Cohort acronym	Cohort name	Main objectives
ABCD	Amsterdam Born Children and Development study	Examining the maternal lifestyle, medical status, psychosocial factors, and environmental conditions during pregnancy, with a particular emphasis on ethnic variations and their impact on the health of children. Key perinatal risk factors encompass nutritional status, psychosocial stress, substance use, medical issues, and exposure to air pollution. The primary areas of focus for outcomes include birth results, infant growth, obesity, metabolic syndrome, cognitive development, psychosocial well-being, and nutritional aspects.
ACCESS	Asthma Coalition on Community, Environment and Social Stress project	Investigating the impact of early-life stress and physical environmental factors on the risk of childhood asthma in urban settings. The primary objective is to examine the pathways connecting social structures at both individual and community levels to health disparities, utilizing an interdisciplinary approach.
ALSPAC	Avon Longitudinal Study of Parents and Children	Broadly defined aims: To explore the connections among genetic, environmental, social, psychological, epigenetic, and biological influences and their impact on the development, overall health, and social outcomes of offspring.
AOF	All Our Families community-based cohort	Broadly defined aims: To examine the well-being during pregnancy and the subsequent outcomes for children and families. Factors under consideration encompass social support, the mental and physical health of mothers, experiences of abuse, lifestyle, utilization of community services, and the accessibility of healthcare services. The outcomes being assessed include child development, overall well-being, and more. This approach employs a lifecourse perspective to comprehend how early-life experiences shape health across the lifespan.
APrON	Alberta Pregnancy Outcomes and Nutrition study	To examine the correlation between maternal nutritional status during pregnancy and the mental well-being of mothers, as well as the health and development of the child.
C-ABCS	China-Anhui Birth Cohort Study	To investigate the postponed, cumulative, and interactive impacts of environmental exposures on mothers on both birth outcomes and the development of children.

Cohort acronym	Cohort name	Main objectives
C-MaMiE	Child outcomes in relation to MAternal Mental health in Ethiopia	To explore common mental disorders in mothers and their impact on the educational and cognitive outcomes of offspring.
CANDLE	The Conditions Affecting Neurocognitive Development and Learning in Early Childhood study	- To assess the impact of in-utero exposure to environmental toxins on birth outcomes and early childhood neurocognitive development.<br>- To investigate the influence of prenatal and infant nutrition on cognitive function in the first years of life.<br>- To explore psychosocial factors and mother-child interactions, evaluating their effect on cognitive development over time.<br>- To identify genetic variants contributing to mothers' and children' responses to nutrition and environmental factors, impacting birth weight and neurocognitive development.
CCREOH	Caribbean Consortium for Research in Environmental and Occupational Health Cohort Study	To explore the impact of both chemical and non-chemical environmental exposures on mothers and their children, emphasizing early childhood neurodevelopment.
CHAMACOS	Center for the Health Assessment of Mothers and Children of Salinas study	To examine pesticides and various environmental exposures within a farmworker community and their impact on the growth, health, and development of children.
DNBC	The Danish National Birth Cohort	Broadly defined: To investigate the link between early-life exposures and disease later on.

Cohort acronym	Cohort name	Main objectives
EDEN	EDEN Mother-Child Cohort Study	To investigate the connections and potential interactions among maternal exposures and health status during pregnancy, fetal development, the infant's health at birth, and the subsequent health and development of the child.
EDMD	Early Determinants of Mammographic Density Study	To examine the correlation between early life factors and mammographic density, a robust predictor of future breast cancer risk.
ELSPAC	The European Longitudinal Study of Pregnancy and Childhood	Broadly defined: To examine the impact of biological, psychosocial, economic, and environmental factors on pregnancy, delivery, and the subsequent development and health of the child.
GIC	Gestational Iodine Cohort	To investigate the connection between gestational iodine deficiency and the neurodevelopmental outcomes of offspring.
GUSTO	Growing Up in Singapore Towards healthy Outcomes	To assess the impact of influences during early development on pathways leading to metabolic compromise and changes in body composition.
HDSS-León	The Nicaraguan Health and Demographic Surveillance System	HDSS-León generally serves as a demographic surveillance site monitoring demographic changes of the region.
INMA	INfancia y Medio Ambiente—Environment and Childhood	To explore the impact of prenatal and early life exposures, particularly environmental pollutants in air, water, and maternal diet, on child growth and development.
KOMCHS	Kyushu Okinawa Maternal and Child Health Study	NS: Main exposures measured are nutrition (150 food and beverage items) and smoke exposure.

Cohort acronym	Cohort name	Main objectives
MoBa	The Norwegian Mother and Child Cohort Study	Broadly defined: To identify the causes of serious diseases by estimating specific associations between exposure and outcomes, with a particular focus on children and also considering parents.
MUSP	The Mater-University of Queensland Study of Pregnancy	Broadly defined: focus on the health consequences of socioeconomic disadvantage (although drop-outs were generally of lower socioeconomic status), as well as the association between maternal characteristics and child health and development.
MYPS	The Mothers' and Young People's Study (formerly known as: Maternal Health Study)	To examine the impact of intergenerational trauma and family violence, with a focus on common maternal physical and psychological health problems, as well as the health consequences of family violence for both women and children.
NA	CHILD-SLEEP birth cohort	To assess various biological, developmental, prenatal, and environmental factors influencing childhood sleep development and the interconnected sleeping difficulties within the family.
NA	Generation R Study	To identify early environmental and genetic factors, as well as the causal pathways, associated with normal and abnormal growth, development, and health from fetal life through childhood and young adulthood.
NA	Gomeroi gaaynggal study	To address the disparity in health outcomes and challenge inequalities experienced by Indigenous women.
NA	Healhty habits for two (2005 follow-up)	NS
NA	Project Viva	Initial aim: To investigate the impact of maternal diet and other factors during and after pregnancy on children. Expanded aims over the past two decades focus on various factors such as physical activity, sleep, environmental chemicals, air pollution, stressors, mental health, and more.

Cohort acronym	Cohort name	Main objectives
NA	The Lifeways Cross-Generation Cohort Study	Broadly defined: To evaluate the impact of socio-economic, lifestyle, cross-generational, and health service factors on health and development. The study encompasses data from mothers, fathers, children, and grandparents.
NA	The Mother-Child Cohort in Crete, Greece (Rhea Study)	Broadly defined: To explore nutritional, environmental, and psychosocial factors influencing children's growth and development.
NA	The Vitamin D in Pregnancy cohort study	To investigate the relationship between prenatal maternal vitamin D status and child growth and development, additionally including a wide range of physical and mental health exposures and outcomes.
NA (1)	NA (1)	NA
NA (2)	NA (2)	NA
NA (3)	NA (3)	NA
NA (4)	NA (4)	NA
NA (5)	NA (5)	NA
NA (6)	NA (6)	NA
NA (7)	NA (7)	NA
NA (8)	NA (8)	NA
NA (9)	NA (9)	NA
NA (10)	NA (10)	NA

Cohort acronym	Cohort name	Main objectives
NA (11)	NA (11)	NA
NCPP	National Collaborative Perinatal Project	To investigate perinatal factors and conditions, including: 1) pregnancy conditions, such as infection, trauma, drug reactions, labor and delivery; 2) environmental factors, such as social and economic characteristics, emotional stress, medical care; 3) biological factors, such as age, medical history, immunologic characteristics; 4) genetic background of parents.
NEST	Newborn Epigenetics STudy	To investigate early life environmental exposures and nutrition and their effect on DNA methylation profiles.
OCC	Odense Child Cohort	To identify at-risk children by assessing factors such as physical activity, dietary habits, alcohol consumption, and smoking with a focus on preventive measures to establish connections between biological and chemical substances, diseases, and lifestyle choices.
PEACHES	Programming of Enhanced Adiposity Risk in Childhood Early Screening	To investigate newborns for the risk of developing obesity later in life, emphasizing the importance of early detection and aiming to identify new markers that may indicate an early risk for future obesity.
PELAGIE	Perturbateurs endocriniens, Étude Longitudinale sur les Anomalies de la Grossesse, l'Infertilité et l'Enfance	To investigate exposure to toxic chemicals, including solvents, pesticides, water chlorination by-products, etc., and their effect on the health and developmental outcomes of children and youngsters.
PROGRESS	Programming Research in Obesity, Growth, Environment, and Social Stressors cohort	To examine the effects of prenatal exposures to mixtures of metals, air pollution, and phthalates on childhood executive function and metabolic outcomes.
QF2011	Queensland Flood study	To assess the effect of maternal prenatal stressors (particularly experience of stress, trauma, PTSD in relation to 2011 floods in Australia) on offspring risk for a variety of disorders.

Cohort acronym	Cohort name	Main objectives
Raine	The Western Australian Pregnancy Cohort Study	Broadly defined: research areas cover 14 key special interest group areas including genetics, lifestyle behaviors, environmental factors, allergies, reproduction etc.
RPGEH Pregnancy Cohort	Research Program on Genes, Environment and Health Pregnancy Cohort (pregnancy cohort is a part of the larger study RPGEH)	To identify genetic and environmental factors that affect common diseases, disease risk factors, and response treatment.
SCDS	The Seychelles Child Development Study	To study child development, including assessing prenatal and postnatal mercury exposure, factors that modulate associations between mercury exposure and child development
SEATON	Study of Eczema and Asthma To Observe effect of Nutrition	To examine whether maternal diet during pregnancy affects their children's risk for asthma and allergies.
SGA	The Scandinavian Small-for-Gestational Age pregnancy and birth cohort	1) To examine various factors, including medical, social, environmental, and genetic aspects, associated with fetal growth that could impact the child's physical and mental development during the initial year of life. 2) To evaluate outcomes in children with an emphasis on the child's physical and mental well-being in connection with information gathered during pregnancy and the first year of life.
SLCDS	South London Child Development Study	To explore the relationship between perinatal mental disorders and a range of risk factors and disorders in children.
SMOK	SSRI in pregnant mothers, outcome of the kids' study	To examine the effect of prenatal exposure to SSRIs on children's cognitive, motor, and behavioral outcomes.

Cohort acronym	Cohort name	Main objectives
S-PRESTO	Singapore Preconception Study of Long-Term Maternal and Child Outcomes	Broadly defined: To investigate the impact of nutrition, lifestyle, and maternal mood before and during pregnancy on children's epigenome, as well as other clinically significant outcomes such as gestational duration, fetal growth, metabolic and neural characteristics in children and more.
